# Bariatric surgery for patients with type 2 diabetes mellitus requiring insulin: Clinical outcome and cost-effectiveness analyses

**DOI:** 10.1371/journal.pmed.1003228

**Published:** 2020-12-07

**Authors:** Emma Rose McGlone, Iain Carey, Vladica Veličković, Prem Chana, Kamal Mahawar, Rachel L. Batterham, James Hopkins, Peter Walton, Robin Kinsman, James Byrne, Shaw Somers, David Kerrigan, Vinod Menon, Cynthia Borg, Ahmed Ahmed, Bruno Sgromo, Chandra Cheruvu, Gul Bano, Catherine Leonard, Howard Thom, Carel W le Roux, Marcus Reddy, Richard Welbourn, Peter Small, Omar A. Khan

**Affiliations:** 1 National Bariatric Surgical Registry (NBSR)/British Obesity and Metabolic Surgical Society (BOMSS), Royal College of Surgeons of England, London, United Kingdom; 2 Department of Metabolism, Digestion and Reproduction, Imperial College London, London, United Kingdom; 3 Population Health Research Institute, St George’s Hospital, University of London, London, United Kingdom; 4 Institute of Public Health, Medical Decision Making and Health Technology Assessment, UMIT, Hall in Tirol, Austria; 5 UCL Centre for Obesity Research, Division of Medicine, Rayne Building, University College London, London, United Kingdom; 6 National Institute of Health Research, UCLH Biomedical Research Centre, London, United Kingdom; 7 St George’s Hospital, London, United Kingdom; 8 Medtronic Ltd, Croxley Green Business Park, Hatters Lane, Watford, United Kingdom; 9 Department of Population Health Sciences, Bristol Medical School, University of Bristol, United Kingdom; 10 Diabetes Complications Research Centre, University College Dublin, Dublin, Ireland; Chinese University of Hong Kong, CHINA

## Abstract

**Background:**

Although bariatric surgery is well established as an effective treatment for patients with obesity and type 2 diabetes mellitus (T2DM), there exists reluctance to increase its availability for patients with severe T2DM. The aims of this study were to examine the impact of bariatric surgery on T2DM resolution in patients with obesity and T2DM requiring insulin (T2DM-Ins) using data from a national database and to develop a health economic model to evaluate the cost-effectiveness of surgery in this cohort when compared to best medical treatment (BMT).

**Methods and findings:**

Clinical data from the National Bariatric Surgical Registry (NBSR), a comprehensive database of bariatric surgery in the United Kingdom, were extracted to analyse outcomes of patients with obesity and T2DM-Ins who underwent primary bariatric surgery between 2009 and 2017. Outcomes for this group were combined with data sourced from a comprehensive literature review in order to develop a state-transition microsimulation model to evaluate cost-effectiveness of bariatric surgery versus BMT for patients over a 5-year time horizon. The main outcome measure for the clinical study was insulin cessation at 1-year post-surgery: relative risks (RR) summarising predictive factors were determined, unadjusted, and after adjusting for variables including age, initial body mass index (BMI), duration of T2DM, and weight loss. Main outcome measures for the economic evaluation were total costs, total quality-adjusted life years (QALYs), and incremental cost-effectiveness ratio (ICER) at willingness-to-pay threshold of GBP£20,000.

A total of 2,484 patients were eligible for inclusion, of which 1,847 had 1-year follow-up data (mean age of 51 years, mean initial BMI 47.2 kg/m^2^, and 64% female). 67% of patients no longer required insulin at 1-year postoperatively: these rates persisted for 4 years. Roux-en-Y gastric bypass (RYGB) was associated with a higher rate of insulin cessation (71.7%) than sleeve gastrectomy (SG; 64.5%; RR 0.92, confidence interval (CI) 0.86–0.99) and adjustable gastric band (AGB; 33.6%; RR 0.45, CI 0.34–0.60; *p* < 0.001). When adjusted for percentage total weight loss and demographic variables, insulin cessation following surgery was comparable for RYGB and SG (RR 0.97, CI 0.90–1.04), with AGB having the lowest cessation rates (RR 0.55, CI 0.40–0.74; *p* < 0.001). Over 5 years, bariatric surgery was cost saving compared to BMT (total cost GBP£22,057 versus GBP£26,286 respectively, incremental difference GBP£4,229). This was due to lower treatment costs as well as reduced diabetes-related complications costs and increased health benefits. Limitations of this study include loss to follow-up of patients within the NBSR dataset and that the time horizon for the economic analysis is limited to 5 years. In addition, the study reflects current medical and surgical treatment regimens for this cohort of patients, which may change.

**Conclusions:**

In this study, we observed that in patients with obesity and T2DM-Ins, bariatric surgery was associated with high rates of postoperative cessation of insulin therapy, which is, in turn, a major driver of overall reductions in direct healthcare cost. Our findings suggest that a strategy utilising bariatric surgery for patients with obesity and T2DM-Ins is cost saving to the national healthcare provider (National Health Service (NHS)) over a 5-year time horizon.

## Abstract 2

Emma Rose McGlone and colleagues report the clinical and cost- effectiveness of bariatric surgery for patients with Type 2 diabetes.

## Author summary

**Why was this study done?:**

- Bariatric or weight loss surgery can dramatically improve type 2 diabetes mellitus (T2DM) in patients with obesity, allowing many patients to stop medicines for T2DM completely.
- Unfortunately, there are limited resources for performing bariatric surgery. Patients with severe T2DM, who require daily insulin injections, are at risk of being considered lower priority than those with T2DM managed by tablet medications. This is because some small studies have suggested that T2DM requiring insulin (T2DM-Ins) is too advanced to be reversed by surgery.
- The aims of this study were to see how effective bariatric surgery is in patients with obesity and T2DM-Ins, as well as to determine if performing surgery in this group is cost-effective over a 5-year period.

**What did the researchers do and find?:**

- We used data from a nationwide registry of patients undergoing bariatric surgery in the UK to examine the effect of surgery on patients that require insulin for T2DM. We found that certain types of procedure (Roux en Y gastric bypass (RYGB) and sleeve gastrectomy (SG)) were associated with excellent rates of stopping insulin (approximately two-thirds of patients).
- We then devised an economic model to compare the costs of this type of surgery with the costs of treating patients using optimal medicines for T2DM. We found that performing surgery was less costly and more effective for the national healthcare provider (National Health Service (NHS)), over a 5-year timeline.

**What do these findings mean?:**

- These findings are important because they suggest that patients with obesity and T2DM-Ins are good candidates for bariatric surgery.
- Offering bariatric surgery to such patients could improve their health and provide cost savings to national healthcare payers.

## Introduction

Over the last decade, bariatric surgery has been shown to be an effective treatment for type 2 diabetes mellitus (T2DM) in patients with obesity [1,2]. Surgery is associated with superior improvement in hyperglycaemia as compared to best medical treatment (BMT), an effect that is sustained for at least 5 years [3–6]. The improvement in hyperglycaemia is associated with a reduction in mortality [7,8] and diabetes-related complications [9,10]. Improvement in T2DM following bariatric surgery is mediated by both weight loss–dependent and weight loss–independent mechanisms [11,12].

T2DM is, however, a progressive, heterogenous disorder with a spectrum of severity [13,14]. To date, the major randomised controlled trials investigating the clinical outcomes of bariatric surgery for T2DM have focused predominantly on patients with more recent onset disease not requiring insulin, as opposed to patients with disease of longer duration requiring insulin [3,4]. To date, there has only been one study, a regional registry analysis, focused on patients who require insulin for their T2DM [15]. A number of retrospective studies have analysed the factors associated with successful diabetes remission following bariatric surgery [16–18]. These studies suggest that patients with more severe T2DM (defined as requiring insulin treatment and/or of long duration) are less likely to experience sustained remission of hyperglycaemia following bariatric surgery. These disappointing findings may explain reluctance in some settings towards surgical treatment for patients with obesity and longer standing T2DM requiring insulin (T2DM-Ins): the national commissioning guidelines in Scotland, for example, permit referral for bariatric surgery only for patients with new-onset T2DM (less than 5 years) [19].

In addition to the clinical benefits of bariatric surgery for individual patients with obesity and T2DM, a number of studies have analysed the healthpayer costs of bariatric surgery [20–22]. Although these studies suggest that surgery is cost-effective as compared to medical management, to date no economic evaluations focus on patients with T2DM-Ins. Moreover, many of these existing economic analyses predate the wide-scale adoption of newer antidiabetic medications such as glucagon-like peptide 1 receptor agonists (GLP-1 RA) and sodium-glucose transport protein 2 (SGLT2) inhibitors—medications which have recently changed the landscape of treatment for patients with more severe T2DM [23]. Though effective, these new medications have significant cost implications. There is a need, therefore, to investigate the clinical outcomes of bariatric surgery in a large cohort of patients with T2DM-Ins and evaluate the cost-effectiveness of a surgical strategy in this cohort.

The purposes of this study were two-fold: firstly, to evaluate clinical outcomes following bariatric surgery in patients with obesity and T2DM-Ins using a UK registry dataset which represents the largest cohort published to date; and secondly, to combine the data with other relevant sources to develop a model-based economic evaluation to assess the cost and cost-effectiveness of bariatric surgery versus BMT in this cohort.

## Methods

The clinical study analysis and cost-effectiveness study framework were planned at the time of study conception, although no formal prospective analysis plan was recorded. For the clinical study, this included the variables that would be adjusted for during regression analysis. For the cost-effectiveness analysis, the model was designed, and data inputs agreed prior to running the model: these data inputs would include those derived from previous studies as well as the present clinical study. After the base case model had been run, and in response to peer reviewers’ comments, three alternative scenario analyses were additionally constructed and run: two for different ethnicities and one with an adjusted hypoglycaemia rate in the BMT group (see S1 Text). This study is reported as per the Consolidated Health Economic Evaluation Reporting Standards (CHEERS) guideline (S1 CHEERS Checklist).

### Design of the clinical study

#### Data source and study population

To evaluate the clinical outcomes of patients with obesity and severe T2DM, as well as prognostic factors predictive of clinical outcomes, we extracted data from the National Bariatric Surgical Registry (NBSR). The NBSR is a bespoke database for the prospective collection of demographic, perioperative, and clinical outcome data for patients with obesity undergoing bariatric surgery in the UK and Republic of Ireland [24]. The details of the demographic and clinical data recorded in this database as well as the data quality are detailed in previous publications [25,26]. Fully anonymised data were extracted from the registry from patients that had previously consented to the collection of their data.

Diabetes status in NBSR is recorded preoperatively and at every postoperative visit as follows: no indication of T2DM; impaired glycaemia or impaired glucose tolerance (diet controlled); oral hypoglycaemics only; or insulin treatment (insulin with or without additional hypoglycaemic medications). As insulin use has consistently been identified as a strong negative predictor of remission of T2DM after bariatric surgery [16–18], we focused on patients that were using insulin for T2DM preoperatively (T2DM-Ins).

From the database, we identified all patients with T2DM-Ins who had undergone primary Roux-en-Y gastric bypass (RYGB), sleeve gastrectomy (SG), or adjustable gastric band (AGB) between 1 January 2009 and 31 May 2017. As the majority of patients have several follow-up episodes recorded on NBSR, data were selected as follows: for outcomes at 1 year, only patients with a follow-up appointment between 6- and 24-months post-surgery were included, and data were extracted from the clinic appointment closest to the 1-year point after surgery; for longer-term outcomes, data were extracted from the final recorded clinic appointment, with exclusion of any patients with less than 2 years’ follow-up. For diabetes status over time, all patients with any follow-up visit were included.

### Ethics statement

The data holder NBSR complied with local ethics guidelines and use of this dataset for research purposes conformed with UK legislation and was approved by the Health Research Authority (17/CAG/0023).

### Analytical approach

Data are presented as mean with standard deviation or number with percentage of total in parentheses.

Percent weight loss (%WL) was calculated as percent of total weight lost using the following formula:

%WL=100×(InitialWeight–Follow‐upWeight)/InitialWeight

Percent excess weight loss (%EWL) was calculated based on an optimum body mass index (BMI) of 25 kg/m^2^, using the following formula:

%EWL=100×(InitialWeight–Follow‐upWeight)/(InitialWeight–OptimumWeight)

Comparison of baseline factors and outcomes by procedure was initially carried out by analysis of variance (means), quantile regression (medians), or chi-squared tests as appropriate. Further adjusted comparison of factors predicting insulin cessation following surgery were made using Poisson regression to generate relative risk (RR) ratios, 95% confidence intervals (CIs) and *p*-values using PROC GENMOD, SAS software version 9.4 (SAS Institute Inc., Cary, North Carolina, United States of America). Robust standard errors were estimated accounting for clustering by hospital, and an offset term was included based on follow-up time. For procedure type, RYGB was chosen as the reference category. Baseline factors used for adjustment were age, gender, initial BMI, smoking, number of comorbidities, duration of diabetes, and ethnicity. Additionally, further models adjusted for postoperative change in weight (with initial BMI removed), in order to assess any potential contributions to improvement in T2DM mediated through weight loss–independent mechanisms of the different operations. This analysis was included because it has been demonstrated that some bariatric procedures (such as RYGB) may be associated with greater weight loss–independent improvements in T2DM as compared to others [11,12]. Age and weight-related variables (BMI, weight loss, and excess weight loss) are presented within categories, but additional models were fitted with them as continuous variables.

### Design of the health economic model

We developed a model-based management protocol in order to compare the costs and effects of a strategy of surgical intervention versus BMT for patients with obesity and T2DM-Ins, over a 5-year timeline horizon. As detailed in full in S1 Text, individual patients with T2DM-Ins were simulated based on characteristics from the UK NBSR dataset and then duplicated to create an identical clone. In the model, one clone was treated with BMT, while the other was treated with bariatric surgery. This strategy ensures that the treatment comparisons are not being influenced by differences in patient characteristics but only based on the treatments received. Each clone or “patient” was then put through the model, and the costs and health outcomes were amassed for all patients in each treatment arm. The model is developed as a state-transition patient-level simulation with 1-year cycle length.

### Model inputs

The data from the NBSR as presented in this paper were utilised to inform baseline patient characteristics for the population (see “Model Inputs,” S1 Text).

For the purposes of this evaluation, given the inferior efficacy of AGB when compared to RYGB and SG in T2DM improvement (see results below), we modelled that patients in the surgical group would only undergo either RYGB or SG. We assumed, based on recent UK procedure prevalence data [27], that 58% would undergo RYGB and 42% SG.

The BMT regimen (which includes nutritional counselling) was determined from the latest guidelines from the American Diabetes Association and European Association for Study of Diabetes, and expert consensus (see S1 Text) [23]. All costs and outcomes after the first year were discounted 3.5% per year in line with National Institute for Health and Care Excellence (NICE) recommendations.

The model is fully described in S1 Text, including the sources from which all model inputs were derived.

### Model outcomes

The model calculates the following outcomes:

1. Total costs (consisting of treatment acquisition costs, cost of adverse events, and cost of disease-related complications);
2. Life-years gained;
3. Quality-adjusted life year (QALY), an outcome that captures life expectancy and quality of life in one measure, and cost per QALY;
4. Incremental costs: the difference in the total cost between bariatric surgery and BMT;
5. Incremental QALYs: the difference in the total QALYs gained between bariatric surgery and BMT;
6. Incremental cost-effectiveness ratio (ICER), which is calculated by dividing the difference in cost between two arms (incremental cost) by the difference in QALYs (incremental QALYs). In general, the treatment option is considered cost-effective when the ICER is below a willingness-to-pay threshold of GBP£20,000/QALY [28];
7. Net monetary benefit (NMB): the value of bariatric surgery in monetary terms under the willingness-to-pay threshold of GBP£20,000/QALY for a unit of benefit. It is calculated as NMB = Δ QALY*λ − Δ cost, where λ is willingness-to-pay threshold in England (GBP£20,000/QALY). Intervention is considered cost-effective when NMB is greater than 0;
8. Net health benefit (NHB): the value of bariatric surgery in terms of health benefit corrected for the incremental costs divided by willingness-to-pay threshold. NHB is calculated as NHB = Δ QALY − Δ cost/λ. Treatment is considered cost-effective when NHB is greater than 0. Both NMB and NHB are presented at willingness-to-pay thresholds of GBP£20,000/QALY.
9. Probabilistic sensitivity analysis–cost-effectiveness plane, which reports an average ICER with a 95% CI and the probability that the intervention is cost-effective.

## Results

### Clinical study

#### Description of cohort

**Fig 1:**
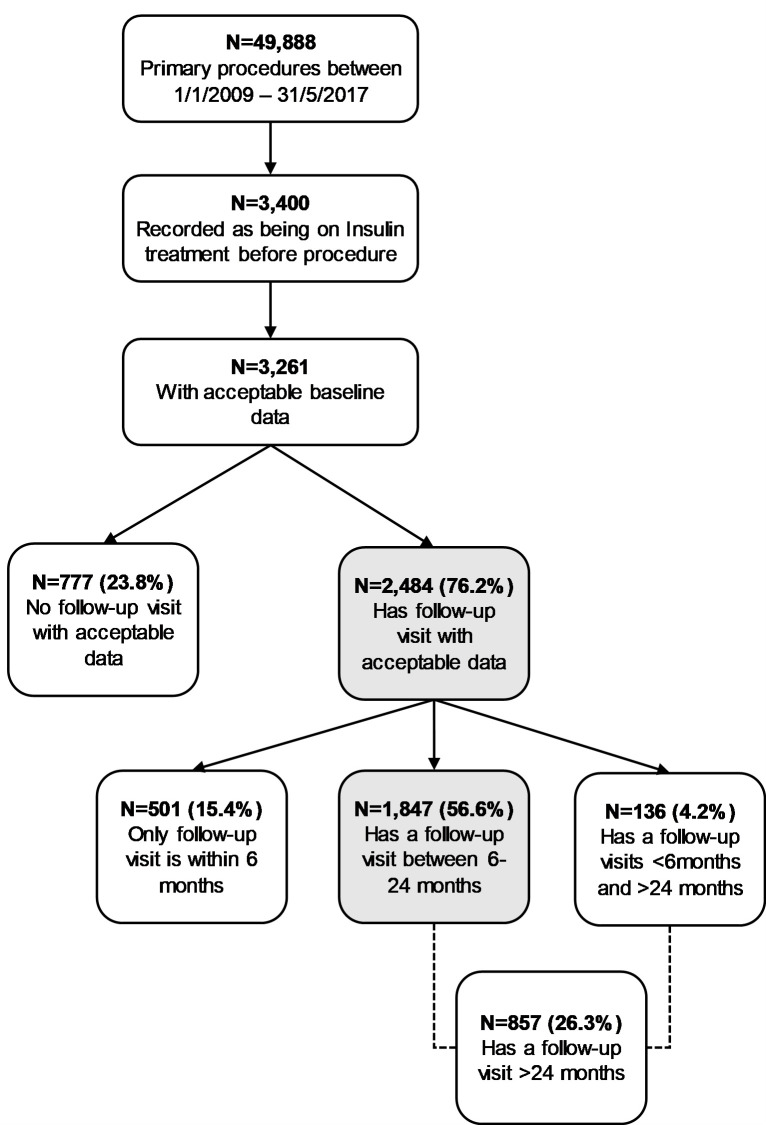
Flowchart to illustrate selection of surgical patients from NBSR database for the clinical study. NBSR, National Bariatric Surgical Registry.

A total of 3,261 surgical patients with T2DM-Ins were identified from the NBSR as having had primary RYGB, SG, or AGB during the designated time frame (Fig 1). Of these, 2,484 (76.2%) had at least 1 follow-up visit recorded in the NSBR, with 1,847 having one visit between 6- and 24-months post-surgery (“1-year follow-up”). Of these, 1,313 (71.1%) underwent RYGB, 397 (21.5%) SG, and 137 (7.4%) AGB. Demographic data for these patients are summarised in Table 1.

Primary procedures were defined as RYBG, SG, or AGB. Acceptable data required all the following: nonzero age, initial weight 70 to 400 kg, height 1 to 2 m, and duration of T2DM recorded.

**Table 1:** Baseline demographic data for surgical patients by procedure (*n* = 1,847).

		All	AGB	SG	RYGB
Number of patients	*n* (%)	1,847 (100)	137 (7.4)	397 (21.5)	1,313 (71.1)
Age (years)	Mean (SD)	51.1 (9.4)	52.6 (9.6)	52.2 (10.1)	50.7 (9.1)
Initial weight (kg)	Mean (SD)	132.7 (24.5)	130.1 (21.8)	138.3 (28.8)	131.2 (23.0)
	Median (IQR)	131.0 (115.3–148.1)	129.0 (113.8–143.6)	134.7 (119.0–153.6)	129.9 (115.0–145.8)
Initial BMI (kg/m^2^)	Mean (SD)	47.2 (7.3)	47.0 (6.3)	49.1 (8.7)	46.6 (6.9)
	Median (IQR)	46.3 (41.8–51.5)	46.9 (41.8–51.5)	47.7 (42.8–54.4)	46.1 (41.6–50.7)
Sex					
Men	*n* (%)	667 (36.1)	44 (32.1)	156 (39.3)	467 (35.6)
Smoking*					
Never	*n* (%)	984 (56.1)	62 (49.6)	238 (63.0)	684 (54.7)
Ex	*n* (%)	642 (36.6)	51 (40.8)	116 (30.7)	475 (38.0)
Current	*n* (%)	127 (7.2)	12 (9.6)	24 (6.4)	91 (7.3)
Duration of T2DM					
0–5 years	*n* (%)	454 (24.6)	29 (21.2)	100 (25.2)	325 (24.8)
6–10 years	*n* (%)	568 (30.8)	42 (30.7)	116 (29.2)	410 (31.2)
>10 years	*n* (%)	825 (44.7)	66 (48.2)	181 (45.6)	578 (44.0)
Number of comorbidities					
0	*n* (%)	525 (28.4)	42 (30.7)	112 (28.2)	371 (28.3)
1	*n* (%)	623 (34.2)	45 (32.9)	133 (33.5)	454 (34.6)
2 or more	*n* (%)	690 (37.4)	50 (36.5)	152 (38.3)	488 (37.2)
Ethnicity*					
White	*n* (%)	1,591 (90.4)	125 (94.0)	328 (87.0)	1,138 (91.0)
Nonwhite	*n* (%)	169 (9.6)	8 (6.0)	49 (13.0)	112 (9.1)

*Expressed among those with a recording in each category. Missing data for the following categories: *n* = 94 (5.1%) had no smoking data record; *n* = 87 (4.7%) had no ethnicity recorded.

AGB, adjustable gastric band; BMI, body mass index; IQR, interquartile range; RYGB, Roux-en-Y gastric bypass; SD, standard deviation; SG, sleeve gastrectomy; T2DM, type 2 diabetes mellitus.

#### Clinical outcomes

At 1-year follow-up (mean of 355 days post-procedure), the mean percentage weight loss was 27.4%, with evidence of differential weight loss between surgical groups (*p* < 0.001; Table 2).

**Table 2:** Outcomes for surgical patients at 1-year follow-up by procedure (*n* = 1,847).

		All (*n* = 1,847)	AGB (*n* = 137)	SG (*n* = 397)	RYGB (*n* = 1,313)	*p*-value**
Days since procedure	Mean (SD)	354.7 (89.1)	362.6 (80.0)	351.2 (91.5)	354.9 (89.2)	0.43
Readmission within 30 days*						
No	*n* (%)	1,718 (94.5)	127 (94.8)	374 (95.4)	1,217 (94.2)	0.64
Yes	*n* (%)	100 (5.5)	7 (5.2)	18 (4.6)	75 (5.8)	
Weight loss						
%WL	Mean (SD)	27.4 (9.3)	15.8 (9.0)	25.1 (9.2)	29.4 (8.2)	<0.001
	Median (IQR)	27.6 (21.6–33.6)	15.5 (10.3–21.3)	24.9 (19.3–30.4)	29.5 (24.1–34.8)	<0.001
%EWL	Mean (SD)	61.5 (22.9)	35.0 (20.1)	54.7 (22.8)	66.3 (20.8)	<0.001
	Median (IQR)	60.6 (47.0–75.3)	34.6 (21.8–49.7)	53.0 (39.8–67.4)	64.9 (52.7–78.7)	<0.001
Diabetes status						
No indication of T2DM	*n* (%)	619 (33.5)	7 (5.1)	119 (30.0)	493 (37.6)	<0.001
Impaired fasting glycaemia	*n* (%)	77 (4.2)	4 (2.9)	19 (4.8)	54 (4.1)	
Oral hypoglycaemics	*n* (%)	548 (29.7)	35 (25.6)	118 (29.7)	395 (30.1)	
Insulin	*n* (%)	603 (32.7)	91 (66.4)	141 (35.5)	371 (28.3)	

*Missing data for *n* = 29 (1.6%) patients

**p-value for tests of heterogeneity between procedure type (ANOVA, comparison of medians using quantile regression, or chi-squared test).

%EWL, percent excess weight loss; %WL, percent weight loss; AGB, adjustable gastric band; ANOVA, analysis of variance; IQR, interquartile range; RYGB, Roux-en-Y gastric bypass; SD, standard deviation; SG, sleeve gastrectomy; T2DM, type 2 diabetes mellitus.

Overall, approximately one-third (32.7%) of the total cohort were still using insulin at 1 year, with another third (33.5%) no longer recorded as having T2DM. There was significant variation in T2DM status by procedure (*p* < 0.001); with 33.6% having ceased use of insulin in the AGB group compared to 64.5% and 71.7% in the SG and RYGB groups, respectively, and a smaller proportion of patients assessed as having no indication of T2DM following AGB (5.1%) than SG and RYGB (30.0% and 37.6%, respectively).

Follow-up of over 2 years (mean 1,132 days, maximum 3,274 days) was available for 857 patients, of whom 605 (70.6%) underwent RYGB, 156 (18.2%) SG, and 96 (11.2%) AGB (Table 3). Weight loss again varied by procedure (*p* < 0.001) with levels broadly similar to those reported at 1-year follow-up. For diabetes status, differences between procedures were still apparent (*p* < 0.001), with a wider gap now seen between the percentage of patients with no indication of T2DM in the RYGB (42.2%) and SG (26.9%) groups.

**Table 3:** Outcomes for surgical patients at final follow-up by procedure (*n* = 857).

		All	AGB	SG	RYGB	*p*-value*
Number of patients	*n* (%)	857 (100)	96 (11.2)	156 (18.2)	605 (70.6)	
Days since procedure	Mean (SD)	1,131.5 (466.9)	1,455.2 (551.3)	1,062.8 (413.6)	1,097.9 (445.3)	<0.001
Weight loss						
%WL	Mean (SD)	27.1 (11.1)	17.2 (10.0)	24.7 (11.5)	29.3 (10.1)	<0.001
	Median (IQR)	27.3 (19.5–34.9)	17.0 (10.8–24.2)	23.5 (17.4–32.2)	29.1 (22.6–36.3)	<0.001
%EWL	Mean (SD)	59.7 (25.0)	37.7 (22.6)	53.3 (26.1)	64.9 (22.8)	<0.001
	Median (IQR)	59.3 (43.6–76.6)	36.5 (24.3–51.0)	51.0 (35.7–70.1)	64.4 (49.8–80.2)	<0.001
Diabetes status						
No indication of T2DM	*n* (%)	314 (36.6)	17 (17.7)	42 (26.9)	255 (42.2)	<0.001
Impaired fasting glycaemia	*n* (%)	40 (4.7)	3 (3.1)	10 (6.4)	27 (4.5)	
Oral hypoglycaemics	*n* (%)	270 (31.5)	24 (25.0)	50 (32.1)	196 (32.4)	
Insulin	*n* (%)	233 (27.2)	52 (54.2)	54 (34.6)	127 (21.0)	

*p-value for tests of heterogeneity between procedure type (ANOVA, comparison of medians using quantile regression, or chi-squared test).

%EWL, percent excess weight loss; %WL, percent weight loss; AGB, adjustable gastric band; ANOVA, analysis of variance; IQR, interquartile range; RYGB, Roux-en-Y gastric bypass; SD, standard deviation; SG, sleeve gastrectomy; T2DM, type 2 diabetes mellitus.

**Fig 2:**
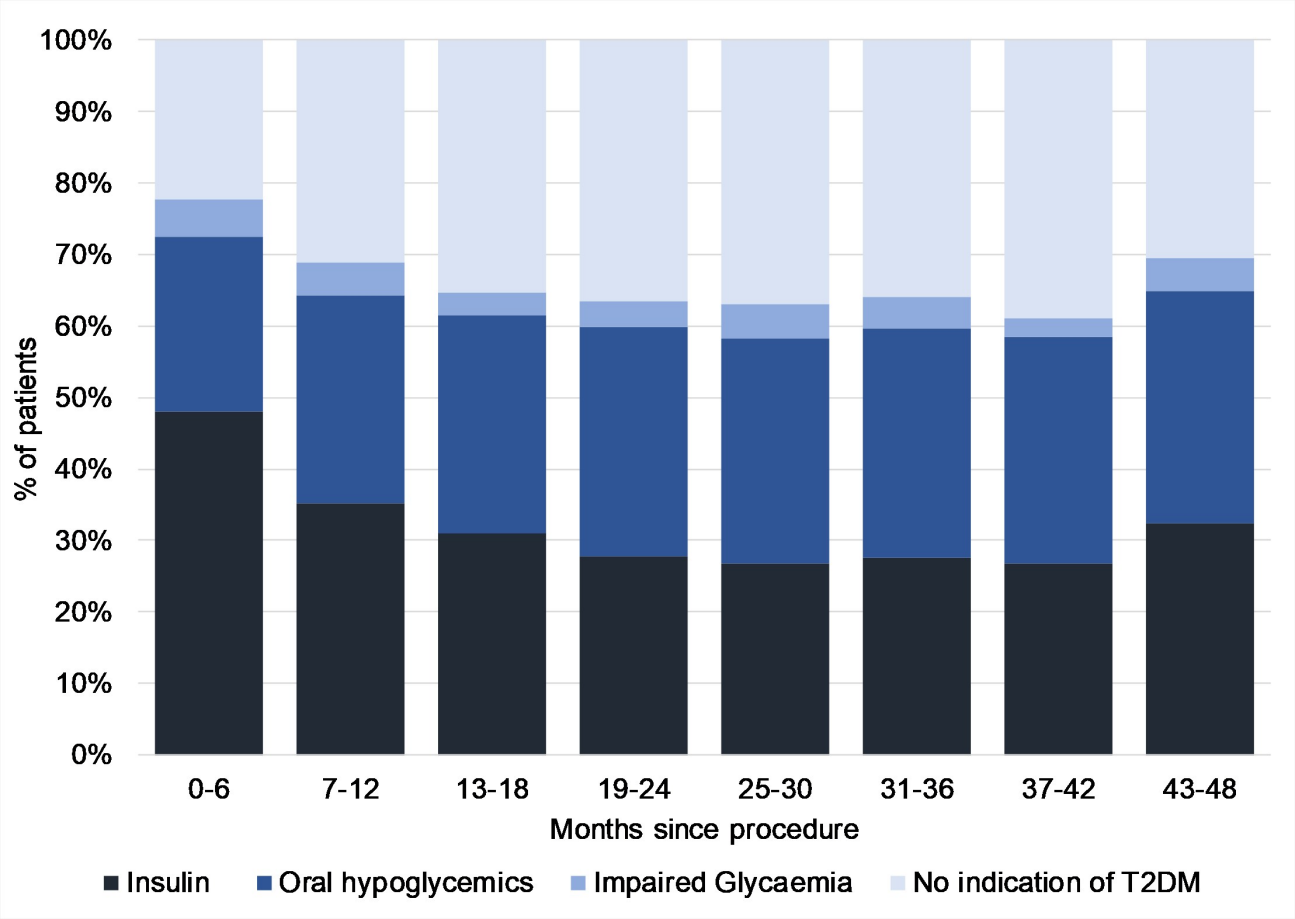
T2DM status in patients over time since bariatric procedure. Percentage of patients with each T2DM status among those with a follow-up visit during each time period from bariatric procedure. Numbers at each visit were the following: month 0 to 6 *n* = 2,150; month 7 to 12 *n* = 1,446; month 13 to 18 *n* = 1,114; month 19 to 24 *n* = 639; month 25 to 30 *n* = 633; month 31 to 36 *n* = 251; month 37 to 42 *n* = 190; month 43 to 48 *n* = 108. T2DM, type 2 diabetes mellitus.

**Fig 3:**
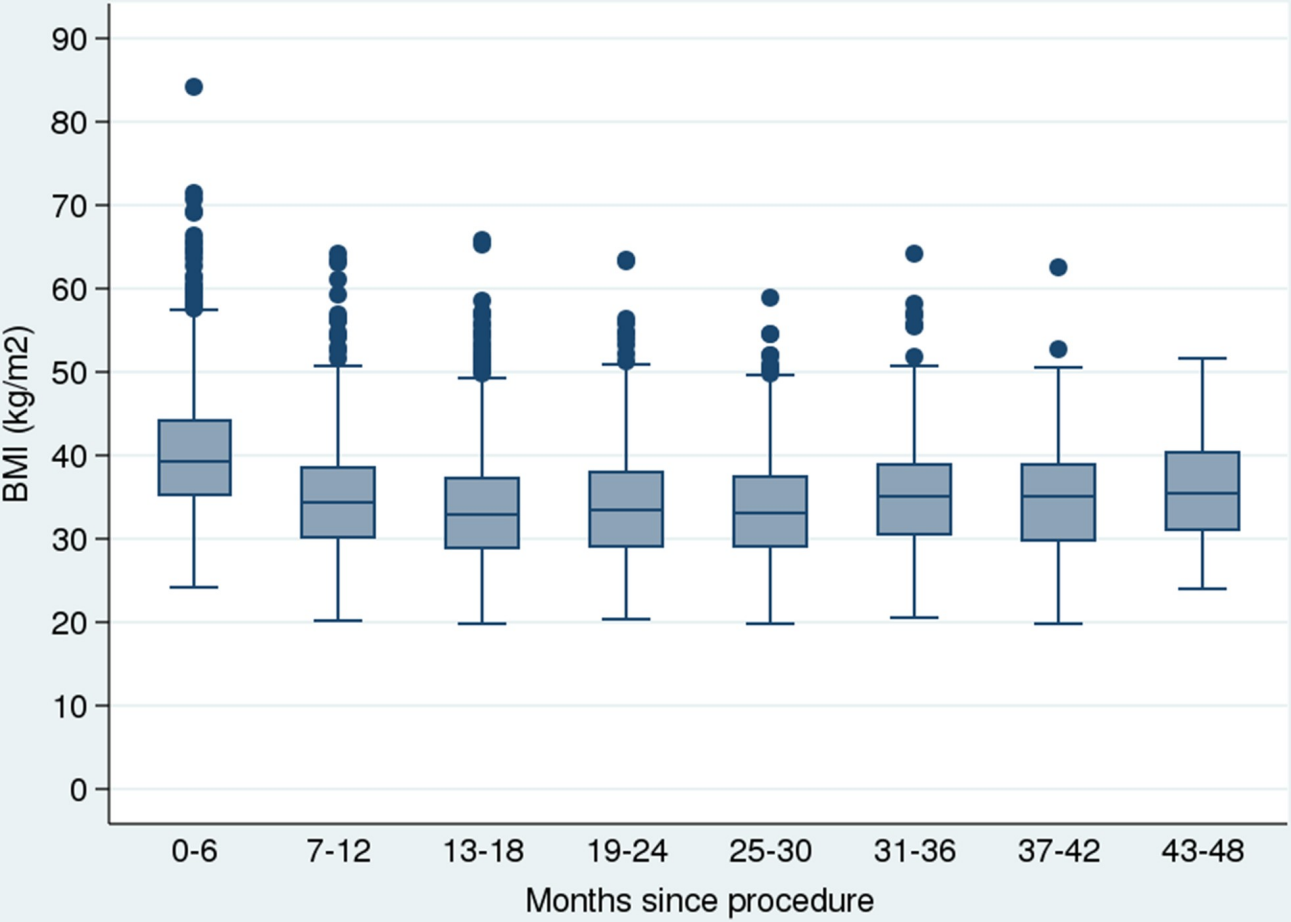
BMI in patients over time since bariatric procedure. Box and whisker plot (median and IQR) to illustrate BMI of patients among those with a follow-up visit during each time period from bariatric procedure. Whiskers represent the upper and lower adjacent values, with dots showing observations beyond these values. Numbers at each visit were the following: month 0 to 6 *n* = 2,133; month 7 to 12 *n* = 1,442; month 13 to 18 *n* = 1,112; month 19 to 24 *n* = 637; month 25 to 30 *n* = 633; month 31 to 36 *n* = 250; month 37 to 42 *n* = 189; month 43 to 48 *n* = 108. BMI, body mass index; IQR, interquartile range.

Fig 2 summarises T2DM status over 4 years, in 3-month periods, for all surgical patients with follow-up within those periods only. Prevalence of insulin use reached a plateau at around 19 to 24 months, stabilising at this level up to 4 years after surgery. Similarly, BMI reached a plateau at around 13 to 18 months, stabilising over the next 4 years (Fig 3).

#### Prediction of insulin cessation by baseline factors

Insulin cessation was more prevalent in patients undergoing RYGB than in patients undergoing SG (RR 0.92, 95% CI 0.86 to 0.99, *p* = 0.02) or AGB (RR 0.45, 95% CI 0.34 to 0.60, *p* < 0.001) (Table 4). Male patients were more likely to cease insulin use after surgery (RR 1.14, 95% CI 1.07 to 1.23, *p* < 0.001). There was no evidence of different insulin cessation rates by number of comorbidities (RR 1.02, 95% 0.94 to 1.11 for 2 or more versus none). Patients with shorter T2DM duration were more likely to cease insulin (RR 1.40, 95% CI 1.29 to 1.53, *p* < 0.001, for patients with duration <5 years as compared to those with duration >10 years). When adjusted for other baseline demographic factors, procedure, gender, and duration of diabetes were all independent predictors of cessation of insulin use after surgery (*p* < 0.001).

**Table 4:** Unadjusted and adjusted RRs for insulin cessation at follow-up by baseline factors (*n* = 1,847).

	*n*	% on insulin at 6–24 month follow-up	RR (95% CI) unadjusted	RR (95% CI) mutually adjusted
Operation type				
AGB	137	66.4%	0.45 (0.34–0.60)	0.46 (0.35–0.61)
RYGB	1,313	28.3%	1	1
SG	397	35.5%	0.92 (0.86–0.99)	0.91 (0.84–0.98)
*p*-value*			<0.001	<0.001
Age (years)				
17–39	222	35.6%	0.89 (0.81–0.98)	0.90 (0.83–0.97)
40–49	540	30.4%	1	1
50–59	718	31.9%	0.95 (0.87–1.04)	0.98 (0.90–1.06)
60+	367	35.7%	0.91 (0.81–1.01)	0.97 (0.88–1.07)
*p*-value^†^			0.96	0.13
Sex				
Female	1,180	35.9%	1	1
Male	667	26.8%	1.14 (1.07–1.23)	1.14 (1.06–1.23)
*p*-value*			<0.001	<0.001
Initial BMI				
40 or less	284	34.5%	1.04 (0.89–1.22)	1.02 (0.87–1.19)
40–45	479	35.3%	1	1
45–50	490	32.0%	1.04 (0.95–1.13)	1.02 (0.94–1.11)
50–55	324	31.2%	1.05 (0.96–1.14)	1.03 (0.95–1.12)
55–60	176	31.8%	1.09 (0.96–1.23)	1.10 (0.98–1.24)
60 or more	94	23.4%	1.14 (0.99–1.32)	1.11 (0.96–1.27)
*p*-value^†^			0.31	0.31
Smoking				
Never	984	32.4%	1	1
Ex vs. never	642	32.9%	1.01 (0.94–1.08)	1.00 (0.93–1.07)
Current vs. never	127	25.2%	1.12 (1.01–1.23)	1.09 (0.98–1.27)
*p*-value*			0.051	0.10
Comorbidities				
0	525	36.2%	1	1
1	632	30.2%	1.06 (0.98–1.16)	1.07 (1.00–1.15)
2 or more	690	32.2%	1.02 (0.94–1.11)	1.03 (0.95–1.12)
*p*-value*			0.70	0.50
T2DM duration (years)				
0–5	454	19.8%	1.40 (1.29–1.53)	1.39 (1.28–1.50)
6–10	568	27.3%	1.31 (1.19–1.44)	1.29 (1.17–1.42)
>10 years	825	43.4%	1	1
*p*-value*			<0.001	<0.001
Ethnicity				
White	1,591	32.4%	1	1
Nonwhite	169	32.5%	1.06 (0.96–1.18)	1.09 (1.00–1.20)
*p*-value*			0.40	0.21

*p-values test for heterogeneity between categories.

^†^*p*-values test for linear trend derived from model where age and BMI were fitted as continuous variables.

AGB, adjustable gastric band; BMI, body mass index; CI, confidence interval; RR, relative risk; RYGB, Roux-en-Y gastric bypass; SG, sleeve gastrectomy; T2DM, type 2 diabetes mellitus.

#### Prediction of insulin cessation by subsequent change in weight

Lower %WL and %EWL at follow-up was associated with significantly poorer rates of insulin cessation (Table 5). To investigate whether the relative improvement in insulin cessation outcomes conferred by RYGB was related to weight loss, the model was fitted adjusting for all baseline factors except initial BMI, with either %WL (WL model) or %EWL (EWL model) included. Once weight loss was adjusted for using either model, SG was no longer associated with statistically inferior rates of insulin cessation compared to RYGB (RR 0.97, 95% CI 0.90 to 1.04, *p* = 0.37 for WL model and RR 0.97, 95% CI 0.91 to 1.04, *p* = 0.44 for EWL model). Even when adjusted for weight loss, however, AGB was still associated with poorer rates of insulin cessation than RYGB (RR 0.55, 95% CI 0.40 to 0.74 for WL model and RR 0.56, 95% CI 0.42 to 0.73 for EWL model, *p* < 0.001).

**Table 5:** Unadjusted and adjusted RRs for insulin cessation at follow-up by subsequent change in weight and procedure (*n* = 1,847).

	*n*	% on insulin at 6–24 month follow-up	RR (95% CI) unadjusted	RR (95% CI) fully adjusted* (WL model)	RR (95% CI) fully adjusted* (EWL model)
Operation type					
AGB	137	66.4%	0.45 (0.34–0.60)	0.55 (0.40–0.74)	0.56 (0.42–0.73)
RYGB	1,313	28.3%	1	1	1
SG	397	35.5%	0.92 (0.86–0.99)	0.97 (0.90–1.04)	0.97 (0.91–1.04)
*p*-value*			<0.001	<0.001	<0.001
Weight loss (%)					
20% or less	369	51.5%	1	1	_
20%–25%	335	36.4%	1.33 (1.17–1.52)	1.21 (1.06–1.38)	_
25%–30%	407	31.9%	1.45 (1.27–1.66)	1.33 (1.16–1.51)	_
30%–35%	359	23.7%	1.55 (1.36–1.78)	1.37 (1.20–1.57)	_
35% or more	377	20.2%	1.53 (1.34–1.74)	1.35 (1.19–1.54)	_
*p*-value^†^			<0.001	<0.001	
Excess weight loss (%)					
25% or less	75	68.0%	0.51 (0.39–0.67)	_	0.64 (0.51–0.80)
25%–50%	471	40.6%	1	_	1
50%–75%	825	30.7%	1.16 (1.06–1.27)	_	1.13 (1.04–1.22)
75%–100%	388	22.4%	1.26 (1.15–1.39)	_	1.19 (1.09–1.30)
100% or more	88	23.9%	1.22 (1.04–1.43)	_	1.19 (1.00–1.40)
*p*-value^†^			<0.001		<0.001

*p-values test for heterogeneity between categories.

^†^*p*-values test for linear trend derived from model where weight loss and excess weight loss were fitted as continuous variables.

AGB, adjustable gastric band; CI, confidence interval; EWL, excess weight loss; RR, relative risk; RYGB, Roux-en-Y gastric bypass; SG, sleeve gastrectomy; WL, weight loss.

To assess whether nonlinearity between age, %WL, %EWL, and insulin cessation would impact the RR for insulin cessation conferred by the different operations, sensitivity analyses fitted alternative models using quadratic terms. These adjusted models generated approximately the same RRs, consistently finding AGB to be inferior to RYGB and SG for insulin cessation (S1 Table).

### Cost and cost-effectiveness analysis

Detailed results are presented in S1 Text. S2–S15 Tables detail model inputs, S16 Table provides additional results, and S17–S19 Tables give uncertainty and scenario analyses. S1–S3 Figs illustrate model design and patient flow through the models, while S4 and S5 Figs provide additional results. The most important economic-related outcomes are summarised and presented below.

#### Treatment acquisition costs

Table 6 summarises the results in terms of cumulative average treatment acquisition cost per patient over 5 years for surgery and BMT. Every patient in the bariatric surgery arm will follow the bariatric surgery costs (S10 Table), whereby their first-year treatment will have one-off surgery costs and subsequent drug costs. Every patient in the BMT arm will have drug costs as detailed in S11 Table which may vary each year, depending on whether treatment strategy stays the same (if hemoglobin A1c (HbA1c) falls below 8%) or is modified (S5 Table). If a patient dies before the end of the 5-year term (from diabetes-related deaths or other deaths), they will no longer contribute to the acquisition costs or any other costs (see S2 Fig for patient flow).

**Table 6:** Treatment acquisition costs of BS versus BMT.

	BS	BMT	Incremental cost (BS–BMT)
Drug costs (GBP£)	641	10,578	−9,937
Surgery cost (GBP£)	5,544	0	5,544
**Total treatment costs (GBP£)**	6,185	10,578	−4,393

Costs over 5 years.

BMT, best medical treatment; BS, bariatric surgery.

As shown, when compared to BMT, bariatric surgery is predicted to a lead to treatment acquisition cost saving over a 5-year time horizon.

#### Adverse events

In relation to costs directly attributable to adverse events of treatment (i.e., in the surgical arm costs of surgical complications plus adverse drug reactions; and in the BMT group the adverse drug reactions), the cumulative costs for the bariatric surgical group were GBP£1,152, and for the group undergoing BMT, GBP£955. This represents an incremental difference of GBP£197 in favour of BMT over a 5-year period.

#### Reductions in disease-related complications

Based on the assumptions of the effects of BMT and bariatric surgery on modifying HbA1c and BMI (both of which were the two most important predictors of T2DM complications, see S1 Text), bariatric surgery leads to a lower cumulative incidence of diabetes-related complications (Table 7) and consequently lower cost for the management of these complications (Table 8).

**Table 7:** Incidence of diabetes-related complications.

	BS	BMT	Incremental difference (BS–BMT)
IHD (%)	3.17	4.12	−0.95
MI (%)	6.02	6.62	−0.60
CHF (%)	2.36	6.43	−4.07
Stroke (%)	1.63	2.06	−0.43
Amputation (%)	0.29	0.67	−0.38
Renal failure (%)	0.42	0.35	0.07

Cumulative incidence over 5 years.

BMT, best medical treatment; BS, bariatric surgery; CHF, chronic heart failure; IHD, ischaemic heart disease; MI, myocardial infarction.

**Table 8:** Costs of T2DM-related complications.

	BS	BMT	Incremental difference (BS–BMT)
IHD cost (GBP£)	414	542	−128
MI cost (GBP£)	381	362	19
CHF cost (GBP£)	187	491	−304
Stroke cost (GBP£)	283	352	−69
Amputation cost (GBP£)	55	113	−58
Renal failure cost (GBP£)	197	144	52
Other costs* (GBP£)	13,203	12,748	455
**Total cost (GBP£)**	14,720	14,753	−33

Cumulative cost of complications over 5 years.

*Non-complication costs of treating T2DM.

BMT, best medical treatment; BS, bariatric surgery; CHF, chronic heart failure; IHD, ischaemic heart disease; MI, myocardial infarction; T2DM, type 2 diabetes mellitus.

### Summary of total costs

In summary, bariatric surgery is predicted to result in a total cost saving of GBP£4,229 when compared to BMT over a 5-year time horizon (Table 9).

**Table 9:** Total costs of bariatric surgery and BMT.

	BS	BMT	Incremental difference (BS–BMT)
Treatment acquisition costs (GBP£)	6,185	10,578	−4,393
Adverse event costs (GBP£)	1,152	955	197
Cost of complications (GBP£)	14,720	14,753	−33
**Total costs (GBP£)**	22,057	26,286	−4,229

Costs over 5 years.

BMT, best medical treatment; BS, bariatric surgery.

Unlike medication costs, the costs of surgery are incurred at the start of the model and not spread over the 5-year period; hence, at approximately 3.5 years after surgery, the total cost of a patient treated with bariatric surgery equals the total cost of a patient treated with BMT, whereby from that breakeven point, bariatric surgery becomes the cost-saving option.

#### Cost-effectiveness

As shown in Table 10, from a cost-effectiveness perspective, when compared to BMT, bariatric surgery is predicted to lead to a lower cumulative incidence of diabetes-related complications and is consequently associated with improved life expectancy and measures of quality of life.

**Table 10:** Cost-effectiveness of bariatric surgery and BMT.

	BS	BMT	Incremental difference (BS–BMT)	ICER (cost/QALY)	NMB (GBP£)	NHB (QALYs)
Costs (GBP£)	22,057	26,286	−4,229	Dominated by BS*	4,731	0.24
Average QALYs	3.18	3.15	0.03			
Life years gained in 5 years	4.47	4.43	0.04			

*Dominated is a health economic term that describes an intervention which is more beneficial and less costly for patients.

BMT, best medical treatment; BS, bariatric surgery; ICER, incremental cost-effectiveness ratio; NHB, net health benefit; NMB, net monetary benefit; QALY, quality-adjusted life year.

Probabilistic sensitivity analysis demonstrated that bariatric surgery consistently leads to cost savings when compared with BMT and in more than 50% of cases to positive incremental health benefits (S16 Table and S5 Fig).

## Discussion

We used the UK NBSR, a nationwide registry of patients undergoing bariatric surgery, to assess clinical outcomes of surgery for patients with obesity and T2DM-Ins. Our findings were then combined with data from the literature to develop a model to analyse cost-effectiveness of bariatric surgery for this cohort, when compared to BMT. The study demonstrated that bariatric surgery in patients with obesity and T2DM-Ins was associated with good medium-term clinical outcomes and was cost saving when compared to best medical management both in terms of “upfront” treatment costs and avoidance of future costs of complications. In this, the largest clinical series to date, two-thirds of patients with T2DM-Ins at baseline ceased use of insulin after surgery, with one-third ceasing all medication for T2DM. Our data suggest that these improvements persist for at least 4 years following surgery. These results are comparable to those of a regional database study which reported that 27% of patients on insulin at baseline continued to take insulin at 3 years post-RYGB, with 40% off all medication for T2DM [15]. Interestingly, our results are also similar to those reported for patients with both recent and long-standing T2DM: in a randomised controlled trial 5 years post-RYGB or SG, 35% of surgical patients had stopped taking all medications for T2DM [4]. Our findings therefore challenge the popular view that bariatric surgery has limited clinical efficacy for patients with more advanced T2DM [14].

Our data indicate that in patients requiring insulin for T2DM, AGB is associated with lower rates of insulin cessation and remission of T2DM than RYGB and SG. Greater weight loss has been associated with superior T2DM outcomes [16,29], and we found that this holds true for patients with T2DM-Ins. Moreover, even when adjusted for weight loss, AGB was associated with lower rates of insulin cessation when compared to RYGB or SG. These findings support the notion that improvements in T2DM seen after bariatric surgery have both weight loss–dependent and weight loss–independent components [12,30]. We observed a trend towards increased insulin cessation over time in the RYGB group, despite weight stability, suggesting a further possible late weight loss–independent effect of RYGB on T2DM.

The major novel finding of this study is that over a 5-year time horizon, the costs of bariatric surgery are predicted to be lower than those of medical management for patients with T2DM-Ins. Of particular note, the greatest savings stem from direct treatment costs: bariatric surgery is significantly cheaper than the medications that would otherwise be prescribed to patients with severe T2DM over a 5-year period. This is an important finding, given that the majority of previous economic evaluations concluded that the direct treatment costs of surgery exceeded those of BMT [21,31–33]. The difference may be explained by the fact that previous studies either predated the widespread adoption of newer pharmacological agents for diabetes or focused on patients with early T2DM who are often maintained on relatively inexpensive single oral agents [20–22].

In terms of adverse events, our financial allocations for surgical complications over the 5-year period are comparable to other models derived from UK data [20,22]; however, the majority of previous studies evaluating the costs of bariatric surgery versus medical management have not included costings for adverse events related to treatment in the medically treated patients [20,21]. The newer medications in use for T2DM such as GLP-1 RA and SGLT-2 inhibitors have side effect profiles that require medical attention and treatment in a proportion of patients [34,35]. Though these adverse medical events do have cost implications, the adverse events costs for surgical patients are still slightly higher than for patients treated with BMT in this study.

With regard to avoidance of complications, previous economic evaluations have demonstrated contradictory results. In the UK, Gulliford and colleagues demonstrated that although a bariatric surgical strategy in patients with obesity and T2DM had higher expected cost than medical management, the cost of subsequent clinical gains was sufficiently low to make surgery cost-effective to a healthcare payer (GBP£7,129/QALY gained) [20]. By contrast, Borisenko and colleagues found that the avoidance of complications through a surgical strategy in patients with obesity and T2DM lead to direct healthcare savings [22]. This saving, however, occurred over a long time horizon (for female patients 10 years and for male patients over a lifetime). We have demonstrated that not only are there cost savings within the cohort of patients with T2DM-Ins due to avoidance of future complications, but also these savings occur over a very short time horizon of less than 5 years. It is likely that the differences between our study findings and previous analyses are due to the fact that our study was designed to specifically investigate the sub-cohort of patients with T2DM-Ins, for whom BMT involves expensive medications.

Our study has some limitations. The NBSR dataset contains limited long-term follow-up: we attempted to address this issue by mandating a minimum follow-up period. Moreover, it should be noted that loss to follow-up rate in this study is comparable to other national registry studies (e.g., [15]), and there is no evidence of any systematic bias with regard to the outcomes of those who did and did not attend follow-up. Furthermore, it should be noted that the total number of patients with T2DM-Ins with 4-year follow-up in the present study is larger than any previously published study. With regard to the comparison of the individual procedures, this was not a randomised controlled trial, and hence, there were significant size disparities (as well as potentially clinical and demographic differences) in the cohorts of patients undergoing AGB, SG, and RYGB. In addition, the NBSR does not include details on the dosage and number of medications that the patients took before and after surgery; however, it should be noted that the classification of diabetes status in the database is likely to have underestimated the efficacy of surgery as some patients in remission of diabetes are placed on prophylactic metformin [36].

With regard to the health economic model, we accept that the lack of HbA1c data in the NBSR cohort is a significant limitation as it does introduce a degree of uncertainty in our estimates of future diabetes-related complications. In the present study, changes in HbA1c levels post-surgery in our model were inferred from previous studies. Nonetheless, HbA1c levels may be less economically important than changes in medications, particularly given our finding that the direct medication costs in the BMT group (as opposed to changes in disease-specific complication rates) are the most significant factor in the cost saving associated with bariatric surgery. As with all models, ours was unable to include all variables that may impact on overall outcomes. For example, our model was not designed to consider the broader cost implications of weight loss and improvement in T2DM status on occupational productivity. Additionally, we were unable to incorporate the potential differential effects of bariatric surgery as compared to BMT on lifestyle factors such as physical exercise and mental health status, which may, in turn, affect health and cost outcomes more broadly. Finally, our time horizon perspective for cost-effectiveness was deliberately short at 5 years, and therefore there is uncertainty around long-term cost-effectiveness.

It is also worth noting that both medical and surgical treatments for patients with obesity and T2DM evolve over time: this is true of the available medications and operations, as well as the evidence regarding their efficacy and side effects. For example, in the surgical group, we have not analysed the impact of other procedures such as loop gastric bypass as this was infrequently performed at the time the data were collected. With respect to BMT, since the time the model was devised, it has become clear that use of both GLP-1 RA and SGLT2 inhibitors may confer additional benefits for patients with T2DM in terms of reduction of adverse cardiovascular and renal outcomes [37,38]—future analyses would factor this in.

In summary, this study provides evidence that a strategy of treating patients with obesity and T2DM-Ins with SG or RYGB is associated with a significant incidence of diabetes remission and a high incidence of cessation of insulin therapy. Moreover, while previous economic analyses have suggested that a surgical strategy for T2DM provides clinical benefits but with higher up-front cost to the healthcare payer, this study indicates that for patients with T2DM-Ins, the total cost to the health payer is reduced following bariatric surgery as compared to BMT over a 5-year time period. This pattern is seen even when the clinical benefits of bariatric surgery over BMT, in terms of avoidance of future complications, are not considered.

## Supporting information

S1 Text Supplementary methods and results for cost-effectiveness analysis. (DOCX)

S1 CHEERS Checklist (DOCX)

S1 Table Adjusted relative risks for insulin cessation at follow-up by baseline factors in alternative models (*n* = 1,847). *Model adjusts for same covariates as Table 4 but for age now fits the following terms: age, age^2^. ^†^Models adjust for same covariates as Table 5 but for age fits the following terms: age, age^2^ and in WL model fits %WL and %WL2, or in EWL model fits %EWL and %EWL2.
(DOCX)

S2 Table Additional patient baseline characteristics. (DOCX)

S3 Table Treatment effect of bariatric surgery on HbA1c. (DOCX)

S4 Table Treatment effect of bariatric surgery on BMI. (DOCX)

S5 Table BMT regimen. Regimen agreed following discussion and unanimous consensus of panel of expert diabetologists: CWL, RB and GB. DPP4 = dipeptidyl peptidase 4; GLP-1 RA = glucagon like peptide-1 receptor agonist; SGLT2 = sodium glucose transport protein 2.
(DOCX)

S6 Table Treatment effect of BMT on HbA1c. (DOCX)

S7 Table Mid-term bariatric surgery complications. (DOCX)

S8 Table Adverse drug events for surgical patients. *% of patients with reduced drug dose and increase in HbA1c. TC:HDL (total cholesterol: high-density lipoproteins).
(DOCX)

S9 Table Adverse drug events for BMT patients. *% of patients with reduced drug dose and increase in HbA1c. TC:HDL (total cholesterol: high-density lipoproteins).
(DOCX)

S10 Table Treatment acquisition costs in bariatric surgery group. (DOCX)

S11 Table Treatment acquisition costs in BMT group. DPP4 = dipeptidyl peptidase 4; GLP-1 RA = glucagon like peptide-1 receptor agonist; SGLT2 = sodium glucose transport protein 2.
(DOCX)

S12 Table Cost of bariatric surgery complications. (DOCX)

S13 Table Costs of T2DM complications. *In-hospital costs of treating acute stroke. Weighted average of HRG AA35A, AA35B, AA35C, AA35D, AA35E, AA35F ^&^Average of cost for years 2–5. ^^^Non-complication costs of treating T2DM.
(DOCX)

S14 Table Utilities and decrements associated with individual complications of T2DM. (DOCX)

S15 Table Decrements in utility associated with bariatric surgery and body mass index (BMI) category. (DOCX)

S16 Table Additional model results. *% of patients with event over 5 years.
(DOCX)

S17 Table Probabilistic sensitivity analysis results (1,000 iterations). (DOCX)

S18 Table Cost-effectiveness results for Afro-Caribbean population. *% of patients with event over 5 years.
(DOCX)

S19 Table Cost-effectiveness results for Indian-Asian population. *% of patients with event over 5 years.
(DOCX)

S20 Table Cost-effectiveness results when annual rate of hypoglycaemia in BMT group is constant at 2.43% across 5 years. (DOCX)

S1 Fig Arms of economic evaluation study. (TIF)

S2 Fig Cost-effectiveness model flow. (TIF)

S3 Fig Risk equations from the UKPDS Outcomes Model (UKPDS OM). (TIF)

S4 Fig Tornado diagram with most influential parameters in incremental cost between bariatric surgery and BMT. (TIF)

S5 Fig Cost-effectiveness probability plan (PSA 1,000 iterations) for ICER (cost per QALY) bariatric surgery versus BMT. (TIF)

## Abbreviations

%EWL: percent excess weight loss
%WL: percent weight loss
AGB: adjustable gastric band
BMI: body mass index
BMT: best medical treatment
CHEERS: Consolidated Health Economic Evaluation Reporting Standards
CI: confidence interval
GLP-1 RA: glucagon-like peptide 1 receptor agonists
HbA1c: hemoglobin A1c
ICER: incremental cost-effectiveness ratio
NBSR: National Bariatric Surgical Registry
NHB: net health benefit
NICE: National Institute for Health and Care Excellence
NMB: net monetary benefit
QALYs: quality-adjusted life years
RR: relative risk
RYGB: Roux-en-Y gastric bypass
SG: sleeve gastrectomy
SGLT2: sodium-glucose transport protein 2
T2DM: type 2 diabetes mellitus
T2DM-Ins: T2DM requiring insulin

## References

[1] Colquitt JL, Pickett K, Loveman E, Frampton GK. Surgery for weight loss in adults. Cochrane Database Syst Rev. 2014;CD003641 10.1002/14651858.CD003641.pub4 25105982 PMC9028049

[2] Gloy VL, Briel M, Bhatt DL, Kashyap SR, Schauer PR, Mingrone G, et al Bariatric surgery versus non-surgical treatment for obesity: a systematic review and meta-analysis of randomised controlled trials. BMJ. 2013;347:f5934–f5934. 10.1136/bmj.f5934 24149519 PMC3806364

[3] Mingrone G, Panunzi S, De Gaetano A, Guidone C, Iaconelli A, Nanni G, et al Bariatric-metabolic surgery versus conventional medical treatment in obese patients with type 2 diabetes: 5 year follow-up of an open-label, single-centre, randomised controlled trial. Lancet. 2015;386:964–973. 10.1016/S0140-6736(15)00075-6 26369473

[4] Schauer PR, Bhatt DL, Kirwan JP, Wolski K, Aminian A, Brethauer SA, et al Bariatric Surgery versus Intensive Medical Therapy for Diabetes—5-Year Outcomes. N Engl J Med. 2017;376:641–651. 10.1056/NEJMoa1600869 28199805 PMC5451258

[5] Sjöström L. Review of the key results from the Swedish Obese Subjects (SOS) trial—a prospective controlled intervention study of bariatric surgery. J Intern Med. 2013;273:219–234. 10.1111/joim.12012 23163728

[6] Gulliford MC, Booth HP, Reddy M, Charlton J, Fildes A, Prevost AT, et al Effect of Contemporary Bariatric Surgical Procedures on Type 2 Diabetes Remission. A Population-Based Matched Cohort Study. Obes Surg. 2016;26:2308–2315. 10.1007/s11695-016-2103-6 26922184 PMC5018032

[7] Billeter AT, Eichel S, Scheurlen KM, Probst P, Kopf S, Müller-Stich BP. Meta-analysis of metabolic surgery versus medical treatment for macrovascular complications and mortality in patients with type 2 diabetes. Surg Obes Relat Dis. 2019 10.1016/j.soard.2019.04.029 31201113

[8] Adams TD, Gress RE, Smith SC, Halverson RC, Simper SC, Rosamond WD, et al Long-term mortality after gastric bypass surgery. N Engl J Med Massachusetts Medical Society; 2007;357:753–761. 10.1056/NEJMoa066603 17715409

[9] Sjöström L, Peltonen M, Jacobson P, Ahlin S, Andersson-Assarsson J, Anveden Å, et al Association of bariatric surgery with long-term remission of type 2 diabetes and with microvascular and macrovascular complications. JAMA American Medical Association; 2014;311:2297–2304. 10.1001/jama.2014.5988 24915261

[10] Billeter AT, Scheurlen KM, Probst P, Eichel S, Nickel F, Kopf S, et al Meta-analysis of metabolic surgery versus medical treatment for microvascular complications in patients with type 2 diabetes mellitus. Br J Surg. 2018;105:168–181. 10.1002/bjs.10724 29405276

[11] Pories WJ, Swanson MS, MacDonald KG, Long SB, Morris PG, Brown BM, et al Who would have thought it? An operation proves to be the most effective therapy for adult-onset diabetes mellitus. Ann Surg. 1995;222:339–50– discussion 350–2. 10.1097/00000658-199509000-00011 7677463 PMC1234815

[12] Batterham RL, Cummings DE. Mechanisms of Diabetes Improvement Following Bariatric/Metabolic Surgery. Diabetes Care. 2016;39:893–901. 10.2337/dc16-0145 27222547 PMC5864134

[13] Fonseca VA. Defining and characterizing the progression of type 2 diabetes. Diabetes Care. American Diabetes Association;2009;32 Suppl 2:S151–6. 10.2337/dc09-S301 19875543 PMC2811457

[14] Busetto L. Timing of bariatric surgery in people with obesity and diabetes. Ann Transl Med. 2015;3:94 10.3978/j.issn.2305-5839.2015.03.62 26015936 PMC4430740

[15] Lemus R, Karni D, Hong D, Gmora S, Breau R, Anvari M. The impact of bariatric surgery on insulin-treated type 2 diabetes patients. Surg Endosc. 2018;32:990–1001. 10.1007/s00464-017-5777-5 28842774

[16] Still CD, Wood GC, Benotti P, Petrick AT, Gabrielsen J, Strodel WE, et al Preoperative prediction of type 2 diabetes remission after Roux-en-Y gastric bypass surgery: a retrospective cohort study. Lancet Diabetes Endocrinol. 2014;2:38–45. 10.1016/S2213-8587(13)70070-6 24579062 PMC3932625

[17] Ramos-Levi A, Sánchez-Pernaute A, Matía P, Cabrerizo L, Barabash A, Hernandez C, et al Diagnosis of diabetes remission after bariatic surgery may be jeopardized by remission criteria and previous hypoglycemic treatment. Obes Surg. 2013;23:1520–1526. 10.1007/s11695-013-0995-y 23702908

[18] Aron-Wisnewsky J, Sokolovska N, Liu Y, Comaneshter DS, Vinker S, Pecht T, et al The advanced-DiaRem score improves prediction of diabetes remission 1 year post-Roux-en-Y gastric bypass. Diabetologia. 2017;60:1892–1902. 10.1007/s00125-017-4371-7 28733906

[19] NHS Scotland. Obesity treatment [Internet]. [cited 2019 May 14]. Available from: https://www2.gov.scot/Resource/0039/00397605.pdf

[20] Gulliford MC, Charlton J, Prevost T, Booth H, Fildes A, Ashworth M, et al Costs and Outcomes of Increasing Access to Bariatric Surgery: Cohort Study and Cost-Effectiveness Analysis Using Electronic Health Records. Value Health. 2017;20:85–92. 10.1016/j.jval.2016.08.734 28212974 PMC5338873

[21] Hoerger TJ, Zhang P, Segel JE, Kahn HS, Barker LE, Couper S. Cost-effectiveness of bariatric surgery for severely obese adults with diabetes. Diabetes Care. 2010;33:1933–1939. 10.2337/dc10-0554 20805271 PMC2928336

[22] Borisenko O, Lukyanov V, Ahmed AR. Cost-utility analysis of bariatric surgery. Br J Surg. 2018;105:1328–1337. 10.1002/bjs.10857 29667178

[23] Davies MJ, D'Alessio DA, Fradkin J, Kernan WN, Mathieu C, Mingrone G, et al Management of hyperglycaemia in type 2 diabetes, 2018. A consensus report by the American Diabetes Association (ADA) and the European Association for the Study of Diabetes (EASD). Diabetologia. 2018;61:2461–2498. 10.1007/s00125-018-4729-5 30288571

[24] Welbourn R, Sarela A, Small PK, Somers S. The United Kingdom National Bariatric Surgery Registry second registry report. 2014.

[25] Khan OA, McGlone ER, Maynard W, Hopkins J, Dexter S, Finlay I, et al Single-stage conversions from failed gastric band to sleeve gastrectomy versus Roux-en-Y gastric bypass: results from the United Kingdom National Bariatric Surgical Registry. Surg Obes Relat Dis. 2018 10.1016/j.soard.2018.06.017 30077665

[26] Miras AD, Kamocka A, Patel D, Dexter S, Finlay I, Hopkins JC, et al Obesity surgery makes patients healthier and more functional: real world results from the United Kingdom National Bariatric Surgery Registry. Surg Obes Relat Dis. 2018;14:1033–1040. 10.1016/j.soard.2018.02.012 29778650 PMC6097875

[27] Hospital Episode Statistics. Centre H& SC information; 2018–19.

[28] Schwarzer R, Rochau U, Saverno K, Jahn B, Bornschein B, Muehlberger N, et al Systematic overview of cost-effectiveness thresholds in ten countries across four continents. J Comp Eff Res. 2015;4:485–504. 10.2217/cer.15.38 26490020

[29] Blackstone R, Bunt JC, Cortés MC, Sugerman HJ. Type 2 diabetes after gastric bypass: remission in five models using HbA1c, fasting blood glucose, and medication status. Surg Obes Relat Dis. 2012;8:548–555. 10.1016/j.soard.2012.05.005 22721581

[30] Steven S, Hollingsworth KG, Al-Mrabeh A, Avery L, Aribisala B, Caslake M, et al Very Low-Calorie Diet and 6 Months of Weight Stability in Type 2 Diabetes: Pathophysiological Changes in Responders and Nonresponders. Diabetes Care American Diabetes Association; 2016;39:808–815. 10.2337/dc15-1942 27002059

[31] Viratanapanu I, Romyen C, Chaivanijchaya K, Sornphiphatphong S, Kattipatanapong W, Techagumpuch A, et al Cost-Effectiveness Evaluation of Bariatric Surgery for Morbidly Obese with Diabetes Patients in Thailand. J Obes. 2019;2019:5383478–6. 10.1155/2019/5383478 30863633 PMC6377984

[32] Tang Q, Sun Z, Zhang N, Xu G, Song P, Xu L, et al Cost-Effectiveness of Bariatric Surgery for Type 2 Diabetes Mellitus: A Randomized Controlled Trial in China. Medicine (Baltimore). 2016;95:e3522 10.1097/MD.0000000000003522 27196454 PMC4902396

[33] Alsumali A, Eguale T, Bairdain S, Samnaliev M. Cost-Effectiveness Analysis of Bariatric Surgery for Morbid Obesity. Obes Surg. 2018;28:2203–2214. 10.1007/s11695-017-3100-0 29335933

[34] Hsia DS, Grove O, Cefalu WT. An update on sodium-glucose co-transporter-2 inhibitors for the treatment of diabetes mellitus. Curr Opin Endocrinol Diabetes Obes. 2017;24:73–79. 10.1097/MED.0000000000000311 27898586 PMC6028052

[35] Prasad-Reddy L, Isaacs D. A clinical review of GLP-1 receptor agonists: efficacy and safety in diabetes and beyond. Drugs Context. 2015;4:212283–19. 10.7573/dic.212283 26213556 PMC4509428

[36] Abramowicz M, Zuccotti G, Pflomm Jean-Marie. Metformin for Prediabetes (Reprinted from The Medical Letters on Drugs and Therapeutics, vol 58, pg141, 2016). JAMA. 2017;317:1171 10.1001/jama.2016.17844 28324089

[37] Neuen BL, Young T, Heerspink HJL, Neal B, Perkovic V, Billot L, et al SGLT2 inhibitors for the prevention of kidney failure in patients with type 2 diabetes: a systematic review and meta-analysis. Lancet Diabetes Endocrinol. 2019;7:845–854. 10.1016/S2213-8587(19)30256-6 31495651

[38] Hernandez AF, Green JB, Janmohamed S, D'Agostino RB, Granger CB, Jones NP, et al Albiglutide and cardiovascular outcomes in patients with type 2 diabetes and cardiovascular disease (Harmony Outcomes): a double-blind, randomised placebo-controlled trial. Lancet. 2018;392:1519–1529. 10.1016/S0140-6736(18)32261-X 30291013

