# Supplementary material for: Bariatric surgery for patients with type 2 diabetes mellitus requiring insulin: Clinical outcome and cost-effectiveness analyses
Source: PLoS Med. 2020 Dec 7;17(12):e1003228. doi: 10.1371/journal.pmed.1003228 (PMC7721482; doi:10.1371/journal.pmed.1003228)
Supplement: S1 Text — (DOCX) [file pmed.1003228.s001.docx]

**SUPPLEMENTARY METHODS AND RESULTS FOR COST-EFFECTIVENESS ANALYSIS**

Model description

We developed a model-based economic evaluation in order to compare the costs and effects of a strategy of surgical intervention versus best medical treatment (BMT) for patients with obesity and T2DM requiring insulin, over a 5-year timeline horizon.

Patients with T2DM requiring insulin were simulated based on characteristics from the UK National Bariatric Surgery Register (NBSR) dataset as detailed in the main manuscript and then duplicated to create an identical clone. In the model, one clone was treated with BMT while the other was treated with bariatric surgery (S1 Figure). To achieve stability and accuracy 10,000 patients were run per treatment option. A patient-level simulation (first-order Monte Carlo microsimulation) was used because this type of model is well-equipped to evaluate complex diseases such as T2DM, where patients can suffer simultaneous complications [1].

The cycle length used in the model is one year, with a half-cycle correction applied. Model flow is illustrated in S2 Figure. At the start of the model in the first cycle, every new generated patient is updated by patient-level characteristic. Systolic blood pressure, total cholesterol to high density lipoprotein ratio, HbA1c, and smoking status are time-dependent covariates, updated in the model every cycle before the patient experiences an event. In each cycle, the patient is at risk of experiencing one of seven complications or death in the first year following the complication, which may be diabetes-related death or death due to natural causes. These complications are ischemic heart disease, myocardial infarction, chronic heart failure, renal event, stroke, amputation and blindness in one eye: identified by the United Kingdom Prospective Diabetes Study Outcomes Model (UKPDS OM) as the major adverse health related outcomes for patients with T2DM and a foundation for cost-effectiveness analysis [2]. Accumulated cost and quality of life are calculated when the patient dies and when the simulation is finished for all patients, average results per intervention group are summarised and compared between best medical treatment (BMT) and bariatric surgery. The model was developed in Microsoft Excel 2016 (Microsoft Corp., Redmond, Washington, DC, USA).

Model inputs

For patients entering either arm of the model we modelled the following baseline characteristics:

- Age (at start of the model)
- Sex (% male)
- Ethnic group
- Smokers (%)
- Peripheral Vascular Disease (PVD %)
- HbA1c (% at the start of the model)
- Systolic Blood Pressure (SBP)
- Lipids Total: HDL
- Atrial Fibrillation (AF %)
- Body Mass Index (BMI kg/m^2^)
- Duration of T2DM (from diagnosis to model start)

The clinical inputs for these baseline characteristics of patients entering the model were derived from clinical and demographic data from the NBSR as listed in Table 1 of the main manuscript. Peripheral Vascular Disease (PVD %) and atrial fibrillation (AF %) were set to zero because we did not have precise inputs from the NBSR registry.

In addition, the characteristics HbA1c, systolic blood pressure and total cholesterol to high density lipoprotein ratio were deemed necessary for the model. As these data are not available from the NBSR, they were based on epidemiological studies utilising health records of patients with T2DM undergoing bariatric surgery in the UK [3]. S2 Table features the clinical inputs for these additional baseline characteristics of patients at the start of the model (i.e. those not already presented in Table 1 of the main manuscript).

Surgical Arm

For the purposes of this evaluation, we modeled that patients in the surgical group would undergo 58% RYGB and 42% SG based on recent data regarding the current prevalence of these procedures in the UK [4].

Effect of bariatric surgery on HbA1C

The treatment effect of surgery on HbA1c was informed by a recent epidemiological study analysing health records of patients with T2DM undergoing bariatric surgery in the UK (S3 Table) [3].

Effect of bariatric surgery on BMI

Excess weight loss for patients undergoing RYGB and SG in the clinical evaluation was used to estimate BMI change (S4 Table). Based on the results detailed in the main manuscript, the effect of bariatric surgery on BMI was modelled as remaining unchanged between year 2 and year 5 (Figure 3).

Best medical treatment (BMT) arm

The initial inputs in relation to clinical and demographic information were cloned from the surgical arm. BMT includes nutritional counselling as a cornerstone of any treatment; and the drugs used for BMT over the course of five years are detailed in S5 Table. The medical regimen was determined by a panel of UK diabetology experts (CLR, RB and GB) after review of the summary treatment algorithm detailed in the 2018 consensus report by the American Diabetes Association (ADA) and the European Association for the Study of Diabetes (EASD) [5] as well as a prediction of the likely efficacy of the BMT treatment regimen at yearly time points using the methodology detailed below.

*Effect of BMT on HbA1c*

A literature search was conducted in order to validate the HbA1c values for non-surgically treated patients. The literature review was conducted in MEDLINE via PubMed using the following search codes:

("Glycated Hemoglobin A"[Mesh] AND "Glycosylated Hemoglobin" OR HbA1c OR "Glycohemoglobin A" OR "Glycosylated Hemoglobin A1c") AND ("Diabetes Mellitus"[Mesh] OR "Diabetes Mellitus"[tiab] OR diabet*[tiab]) AND ("Obesity"[Mesh] OR obes*) AND ("United Kingdom"[Mesh] OR "Great Britain" OR "Isle of Man" OR England).

To meet the inclusion criteria, the article was required to report dynamic changes of HbA1c over time (minimum 5 years) in patients with T2DM and obesity and to provide granular details about drugs used for therapy and eventually impact of the drug on HbA1c. The search retrieved 145 articles and after restriction to studies published in the last five years, the total number of eligible articles reduced to 67. After screening by title and abstract, 23 articles were included for full-text screening, of which six studies were chosen for inclusion [7-12]. It is evident from these identified studies that, despite the most recent medical treatment options being offered to this group of patients, their HbA1c level remained higher than 8 mmol/l (even in those with a duration of T2DM < 2 years). Taking that into account, the BMT arm of our model has the best-case scenario in terms of HbA1c levels over five years (S6 Table).

*Effect of BMT on BMI*

Although insulin is obesogenic, metformin and other agents used in combination with insulin in the BMT group (S5 Table) either encourage weight loss or are weight neutral [13]. The assumption was therefore made that patients treated with BMT in the model would have a BMI that remained unchanged over the course of five years.

BMT medical therapy regimen (S5 Table)

Based on the demographic information from our clinical study (from which patients entering BMT were cloned), our BMT patients were modelled as suffering from severe obesity and T2DM requiring insulin with relatively poor glycaemic control.  Given we were undertaking a cost-effectiveness analysis, we started with the assumption that these patients would be in the arm of the ADA/EASD algorithm [6] labelled “Cost is a major issue”. We therefore assumed that at the time patients were "enrolled" into the BMT regimen they were already on Metformin and Insulin as per this arm of the algorithm. In year one the intervention for this cohort was the addition of DPP4i, as per this arm of the algorithm.

In year 2 we escalated according to the algorithm arm entitled “Compelling need to promote weight loss”. This algorithm advises the initial use of GLP-1 RA or SGLT2 inhibitors. We chose GLP-1 RA over SGLT2 inhibitors as our initial escalation therapy due to evidence from a randomised controlled trial of the superiority of GLP1-RA over SGLT2 inhibitors in improving HbA1C and bodyweight [6]- a study which one of expert diabetology panel (CLR) was a co-author.

For year 3 we added in a SGLT2 inhibitor as per the second line of this arm of the algorithm on the assumption (as justified above) that although optimisation of the medication regimen would prevent further increases in body weight or Hba1c levels, these parameters would remain significantly elevated. As such this maximally optimised medication regimen was continued to year 5.

Adverse effects

*Surgical Arm*

Complications of surgery are not reported in detail in the NBSR, and therefore for the economic model surgical complications were estimated based on the Swedish Obese Subjects study (SOREG) and are provided in S7 Table [14].

Based on the results of our clinical evaluation of NBSR data, which demonstrated that a proportion of patients post-surgery require on-going medication for T2DM (Tables 2 and 3), the cumulative adverse effects of these medications were modelled as shown in S8 Table.

*BMT arm*

Based on the United Kingdom Prospective Diabetes Study (UKPDS), we estimated the incidence of adverse events related to medication in the BMT group [15] (S9 Table).

Prevalence and costing

In the BMT arm we included the hypoglycemic risk with an incidence of 2.43% in the first year [16], and 0% incidence after year one (S9 Table). In parallel, for the drugs used in the bariatric surgery arm, we gave a hypoglycemic risk of 3.94% for the first three years (S8 Table). Every hypoglycemic event correlated to a one-off treatment cost of GBP 421 (S13 Table), and a small one-off decrement in utility of -0.047. Therefore, the net difference for both costs and quality of life was negative for bariatric surgery.

All other adverse events in the best medical management arm are set to zero, including oedema, hip fracture, non-compliance (and therefore increase in HbA1c and reduction of dosage due to the non-compliance) and weight gain. We allowed negative values for the change in TC/HDL for year one and year three in the BMT arm (S9 Table).

By comparison, in the bariatric surgery arm, apart from drug-related hypolipemic risk, we included all surgical complications reported in SOREG registry, including cholecystectomy, abdominal wall hernia operations, leakage and abscess, stricture, gastric ulcer, and obstruction in line with their incidence in the first two years, as reported in S7 Table. For all patients in the bariatric surgery arm we have applied a disutility of - 0.0175 for surgery itself to approximate a reduction in quality of life due to the complications. The costs of treatment for those complications are reported on S12 Table, offering a differentiation for the first and second year. It is important to underline that all costs incurred in the first year are not discounted and therefore are higher than discounted costs in subsequent years.

Diabetes-related complications

Based on the impact of the two arms on HbA1c and BMI, the model utilises the risk equations from the UKPDS The Core Diabetes Model (UKPDS OM) [15] to model clinical outcomes and diabetes-related complications. S3 Figure reports the respective equations with hazard and odds ratios for each risk factor per equation. The arrows depict the presence and direction of event-related dependencies between equations. For example, the patient who experiences CHF in one model cycle will have, in the next model cycle, an increased (higher than without CHF) risk of myocardial infarction. The order in which risk of events is applied to individual patients is randomised at the start of each cycle to reflect real-world clinical settings. The equations utilised and their detailed explanations have been reported [15].

Costings

All costs in the analysis have been inflated to 2018 values.

*Treatment acquisition costs*

The cost of index surgeries was based on Health Care Resource Groups (HRGs) [4] and analysis was performed using "HRG4+ 2018/19 Reference Costs Grouper" [17].

The cost of drugs for both arms was informed by NHS Electronic Drug Tariff [18]. S10 Table demonstrates the predicted costs for the surgical arm over a 5-year time horizon. S11 Table demonstrates predicted costs for the BMT group over 5 years.

*Costs of surgical complications and adverse drug events*

Costs of surgical complications were determined from the NHS National Schedule of Reference costs and are given in S12 Table [18].

*Direct medical costs of diabetes complications*

Costs of complications of T2DM are given in S13 Table [15,19].

Effectiveness and quality of life inputs

Life expectancy (including life years gained and diabetes related deaths) has the limitation as an effectiveness outcome that it does not capture the quality of life of patients, and small differences in the quality of life between two interventions can have a substantial impact on patients over the time horizon of five years.

The generic quality of life questionnaire (EQ-5D-3L) is commonly used to determine a patient's quality of life in different health conditions [20]. By measuring quality of life (QoL) with EQ-5D-3L, utilities can be derived with values between − 0.594 and 1, where 0 is death and 1 is perfect health, and results less than zero are considered as a worse than death health state. By multiplying utilities with life expectancy, quality adjusted life years (QALYs) can be derived.

In this model, granular utilities and decrements specifically determined for our UK population with T2DM treated with or without bariatric surgery were used. Decrements specific to bariatric surgery and BMI categories were informed by a systematic review of cost-effectiveness models and the UK HTA reports[21-24]. Results are given in S14 and S15 Tables.

Additional model results

In addition to the clinical effectiveness detailed in the main manuscript, additional clinical effectiveness results are presented in S16 Table.

Uncertainty analysis

In order to quantify uncertainty in the health-economic model, data inputs were evaluated in deterministic and probabilistic sensitivity analysis. One-way deterministic sensitivity analysis was conducted by varying one model parameter at the time while keeping all other parameters at the base-case values. The model is automatized to repeat procedure for all parameters, and results are reported as a Tornado diagram for the 20 most influential parameters. Deterministic sensitivity analysis demonstrated that among the model's 20 most influential parameters are drug cost, initial changes to HbA1c values due to the effects on further diabetic complications, as well as utility decrements for amputations and hypoglycaemia and risk for hypoglycaemia (S4 Figure).

Monte Carlo probabilistic sensitivity analysis (PSA) with 1,000 iterations was conducted, where a random value for each variable is drawn based on a pre-determined distribution. The results of the PSA are graphically reported as a cost-effectiveness plane. Probabilistic sensitivity analysis demonstrated that bariatric surgery consistently leads to cost-savings when compared with BMT, and in more than 50% of cases to positive incremental health benefits (S17 Table and S5 Figure).

As reported in S17 Table, bariatric surgery yielded a QALY gain of 3.485 compared to a gain of 3.469 with BMT (mean values), resulting in an incremental difference of 0.0155 in favour of bariatric surgery. Taking into account that 10,000 patients were run per each treatment option in the base case, repeating that analysis with all patients 1,000 times in probabilistic sensitivity analysis produces results that almost cover the entire spectrum of plausible values from input parameters. Using all plausible values of input parameters, in more than 50% of cases bariatric surgery leads to an increase in relative health benefit. For clinicians who are more familiar with the interpretation of the results of the clinical trials, probabilistic sensitivity results from microsimulation model can be a good approximation of a clinical trial where results on the individual level are not always superior toward intervention versus control, thus generating our conclusion based on mean values.

Scenario analyses

As there are several important factors (glycaemic level and other complications) that differ across ethnicities, we have run two scenario analyses separately for Afro-Caribbean and Indian-Asian populations, given that the base-case analyses were conducted for a White population. The model can run one ethnic group at a time, and combinations of different fractions in the one model run are not possible.

The results are presented in S18 and S19 Tables. As expected, there is a difference in the total expected costs and outcomes per arm, but the overall conclusions are the same as in the base-case results.

With respect to the projected incidence of hypoglycaemia for patients in BMT group over the 5-year time period, we have used a conservative estimate in the main model, in which after the first year of drug escalation the rate of hypoglycaemic episodes is predicted to be 0%. An alternative scenario is that the rate of hypoglycaemia remains constant at 2.43% over the 5 years. The results for this alternative scenario analysis are presented in S20 Table: it leads to similar costings outcomes as the base-case scenario.

Model validation

The model validation was conducted in line with guideline recommendations for good methods practice utilising (i) Face validity, (ii) Internal validity, (iii) Cross-validation, (iv) External validation, (v) Predictive validation.

Face validity, in relation to the model’s structure, data sources, problem formulation and results, was performed by our panel of UK metabolic, epidemiology and health economic experts. Besides providing inputs to inform the study design, from conceptualisation to evidence, this expert panel advised on the selection of drugs for best medical treatment arm.

Rigorous internal validity (sometimes depicted as an internal consistency or technical validity) was conducted to test the mathematical calculations and VBA programming code, and to identify and correct all possible technical errors. We used a standardised procedure with an extensive verification list that was checked by two health economists (VV as model developer and HT as independent health economic consultant). In addition, analysis of extremes were conducted, in which extreme model input values were used to check if there is a significant deviation in the model outputs (e.g. setting all cost inputs to zero in both arms should lead to zero cost in the final results; or setting all mortality inputs to zero should result in both arms experiencing life years gained of five years). All identified errors were corrected before producing results.

Cross-validation of our model with others investigating a similar research problem was performed: this is referred to in the discussion section of the main manuscript.

We developed the model in which we nested UKPDS equations (from UKPDS Outcomes Model [2] ) into the state-transition model. UKPDS equations were selected because those equations have extensive internal and external validation among people with diagnosed T2DM for prediction of CVD and other complications. The UKPDS Outcomes Model is based on patient-level data from the United Kingdom Prospective Diabetes Study (UKPDS) with 30 years of follow up. Even in the extreme case when, for this UKPDS cohort of patients, equations have either over- or under-estimated certain complications, that imprecision will be symmetrically inbuilt in the two arms of the model leading to the same incremental difference.

**SUPPLEMENTARY REFERENCES**

1. Siebert U, Alagoz O, Bayoumi AM, Jahn B, Owens DK, Cohen DJ, et al. State-transition modeling: a report of the ISPOR-SMDM Modeling Good Research Practices Task Force--3. Value Health. 2012;15: 812–820. doi:10.1016/j.jval.2012.06.014

2. Clarke PM, Gray AM, Briggs A, Farmer AJ, Fenn P, Stevens RJ, et al. A model to estimate the lifetime health outcomes of patients with type 2 diabetes: the United Kingdom Prospective Diabetes Study (UKPDS) Outcomes Model (UKPDS no. 68). Diabetologia. 2004;47: 1747–1759. doi:10.1007/s00125-004-1527-z

3. Gulliford MC, Booth HP, Reddy M, et al. Effect of Contemporary Bariatric Surgical Procedures on Type 2 Diabetes Remission. A Population-Based Matched Cohort Study. Obes Surg 2016; 26:2308–15. doi:10.1007/s11695-016-2103-6

4. Hospital Episode Statistics. Centre H& SC information; 2018-19. HES data Copyright © 2019 Re-used with the permission of NHS Digital. All rights reserved

5. Davies MJ, D'Alessio DA, Fradkin J, Kernan WN, Mathieu C, Mingrone G, et al. Management of hyperglycaemia in type 2 diabetes, 2018. A consensus report by the American Diabetes Association (ADA) and the European Association for the Study of Diabetes (EASD). Diabetologia. 2018;61: 2461–2498. doi:10.1007/s00125-018-4729-5

6. Lingvay I, Catarig A-M, Frias JP, Kumar H, Lausvig NL, le Roux CW, et al. Efficacy and safety of once-weekly semaglutide versus daily canagliflozin as add-on to metformin in patients with type 2 diabetes (SUSTAIN 8): a double-blind, phase 3b, randomised controlled trial. Lancet Diabetes Endocrinol. 2019;7: 834–844. doi:10.1016/S2213-8587(19)30311-0

7. Dennis JM, Henley WE, Weedon MN, Lonergan M, Rodgers LR, Jones AG, et al. Sex and BMI Alter the Benefits and Risks of Sulfonylureas and Thiazolidinediones in Type 2 Diabetes: A Framework for Evaluating Stratification Using Routine Clinical and Individual Trial Data. Diabetes Care. 2018;41: 1844–1853. doi:10.2337/dc18-0344

8. Anyanwagu U, Mamza J, Mehta R, Donnelly R, Idris I. Cardiovascular events and all-cause mortality with insulin versus glucagon-like peptide-1 analogue in type 2 diabetes. Heart. 2016;102: 1581–1587. doi:10.1136/heartjnl-2015-309164

9. Heald AH, Livingston M, Malipatil N, Becher M, Craig J, Stedman M, et al. Improving type 2 diabetes mellitus glycaemic outcomes is possible without spending more on medication: Lessons from the UK National Diabetes Audit. Diabetes Obes Metab. 2018;20: 185–194. doi:10.1111/dom.13067

10. Heald AH, Fryer AA, Anderson SG, Livingston M, Lunt M, Davies M, et al. Sodium-glucose co-transporter-2 inhibitors, the latest residents on the block: Impact on glycaemic control at a general practice level in England. Diabetes Obes Metab. 2018;20: 1659–1669. doi:10.1111/dom.13281

11. Khunti K, Godec TR, Medina J, Garcia-Alvarez L, Hiller J, Gomes MB, et al. Patterns of glycaemic control in patients with type 2 diabetes mellitus initiating second-line therapy after metformin monotherapy: Retrospective data for 10 256 individuals from the United Kingdom and Germany. Diabetes Obes Metab. 2018;20: 389–399. doi:10.1111/dom.13083

12. Idris I, Gordon J, Tilling C, Vora J. A cost comparison of long-acting insulin analogs vs NPH insulin-based treatment in patients with type 2 diabetes using routinely collected primary care data from the UK. J Med Econ. 2015;18: 273–282. doi:10.3111/13696998.2014.991788

13. Barnett A, Allsworth J, Jameson K, Mann R. A review of the effects of antihyperglycaemic agents on body weight: the potential of incretin targeted therapies. Curr Med Res Opin. 2007;23: 1493–1507. doi:10.1185/030079907X199691

14. Sjöström L, Lindroos A-K, Peltonen M, Torgerson J, Bouchard C, Carlsson B, et al. Lifestyle, diabetes, and cardiovascular risk factors 10 years after bariatric surgery. N Engl J Med. 2004;351: 2683–2693. doi:10.1056/NEJMoa035622

15. Alva ML, Gray A, Mihaylova B, Leal J, Holman RR. The impact of diabetes-related complications on healthcare costs: new results from the UKPDS (UKPDS 84). Diabet Med. 2015;32: 459–466. doi:10.1111/dme.12647

16. Intensive blood-glucose control with sulphonylureas or insulin compared with conventional treatment and risk of complications in patients with type 2 diabetes (UKPDS 33). UK Prospective Diabetes Study (UKPDS) Group. Lancet. 1998;352: 837–853.

17. NHS Workforce Team. HRG4+ 2017/18 Reference Costs Grouper [Internet]. [cited 1 Jul 2019]. Available from: https://digital.nhs.uk/services/national-casemix-office/downloads-groupers-and-tools/costing-hrg4-2017-18-reference-costs-grouper

18. NHS Workforce Team. Reference Costs [Internet]. 11 Oct 2018 [cited 31 Jul 2019]. Available from: https://digital.nhs.uk/data-and-information/data-collections-and-data-sets/data-collections/reference-costs

19. Luengo-Fernandez R, Silver LE, Gutnikov SA, Gray AM, Rothwell PM. Hospitalization resource use and costs before and after TIA and stroke: results from a population-based cohort study (OXVASC). Value Health. 2013;16: 280–287. doi:10.1016/j.jval.2012.10.013

20. Dolan P. Modeling valuations for EuroQol health states. Med Care. 1997;35: 1095–1108.

21. McCormick JT, Papasavas PK, Caushaj PF, Gagné DJ. Laparoscopic revision of failed open bariatric procedures. Surg Endosc. 2003;17: 413–415. doi:10.1007/s00464-002-8533-3

22. Hakim Z, Wolf A, Garrison LP. Estimating the effect of changes in body mass index on health state preferences. Pharmacoeconomics. 2002;20: 393–404. doi:10.2165/00019053-200220060-00004

23. Sullivan PW, Slejko JF, Sculpher MJ, Ghushchyan V. Catalogue of EQ-5D scores for the United Kingdom. Med Decis Making. 2nd ed. 2011;31: 800–804. doi:10.1177/0272989X11401031

24. Sullivan PW, Ghushchyan VH. EQ-5D Scores for Diabetes-Related Comorbidities. Value Health. 2016;19: 1002–1008. doi:10.1016/j.jval.2016.05.018
