# Supplementary material for: Bariatric surgery for patients with type 2 diabetes mellitus requiring insulin: Clinical outcome and cost-effectiveness analyses
Source: PLoS Med. 2020 Dec 7;17(12):e1003228. doi: 10.1371/journal.pmed.1003228 (PMC7721482; doi:10.1371/journal.pmed.1003228)
Supplement: S1 Table — *Model adjusts for same covariates as Table 4 but for age now fits the following terms: age, age2. †Models adjust for same covariates as Table 5 but for age fits the following terms: age, age2 and in WL model fits %WL and %WL2, or in EWL model fits %EWL and %EWL2. (DOCX) [file pmed.1003228.s003.docx]

**S1 Table**: **Adjusted relative risks for insulin cessation at follow-up by baseline factors in alternative models (n=1,847)**

|  | **RR (95% CI)**  **Fully Adjusted***  **(BMI model)** | **RR (95% CI)**  **Fully Adjusted† (WL model)** | **RR (95% CI)**  **Fully Adjusted† (EWL model)** |
| --- | --- | --- | --- |
| Operation Type |  |  |  |
| - AGB | 0.46 (0.35-0.61) | 0.55 (0.41-0.73) | 0.53 (0.40-0.71) |
| - RYGB | 1 | 1 | 1 |
| - SG | 0.91 (0.84-0.98) | 0.97 (0.91-1.04) | 0.98 (0.91-1.06) |
| p-value test for heterogeneity | <0.001 | <0.001 | <0.001 |

* - Model adjusts for same covariates as Table 4 but for age now fits the following terms: age, age^2^

**†** - Models adjust for same covariates as Table 5 but for age fits the following terms: age, age^2^ and in WL model fits %WL and %WL^2^, or in EWL model fits %EWL and %EWL^2^.
