# Supplementary material for: Bariatric surgery for patients with type 2 diabetes mellitus requiring insulin: Clinical outcome and cost-effectiveness analyses
Source: PLoS Med. 2020 Dec 7;17(12):e1003228. doi: 10.1371/journal.pmed.1003228 (PMC7721482; doi:10.1371/journal.pmed.1003228)
Supplement: S2 Table — (DOCX) [file pmed.1003228.s004.docx]

**S2 Table. Additional patient baseline characteristics**

| **Predictor** | **Value** |
| --- | --- |
| HbA1c (start of model) (%) | 8.16 |
| Systolic blood pressure (SBP) (mmHg) | 139.3 |
| Total: HDL lipids | 4.65 |
