# Supplementary material for: Bariatric surgery for patients with type 2 diabetes mellitus requiring insulin: Clinical outcome and cost-effectiveness analyses
Source: PLoS Med. 2020 Dec 7;17(12):e1003228. doi: 10.1371/journal.pmed.1003228 (PMC7721482; doi:10.1371/journal.pmed.1003228)
Supplement: S3 Table — (DOCX) [file pmed.1003228.s005.docx]

**S3 Table. Treatment effect of bariatric surgery on HbA1c**

| **Years from surgery** | **HbA1c (%)** | **Deterministic sensitivity analysis range** | **Probabilistic sensitivity analysis distribution** |
| --- | --- | --- | --- |
| Year 1 | 6.78 | +/-20% | Normal invariant on HbA1c changes from baseline |
| Year 2 | 6.53 |  |  |
| Year 3 | 6.75 |  |  |
| Year 4 | 6.98 |  |  |
| Year 5 | 6.98 |  |  |
