# Supplementary material for: Bariatric surgery for patients with type 2 diabetes mellitus requiring insulin: Clinical outcome and cost-effectiveness analyses
Source: PLoS Med. 2020 Dec 7;17(12):e1003228. doi: 10.1371/journal.pmed.1003228 (PMC7721482; doi:10.1371/journal.pmed.1003228)
Supplement: S4 Table — (DOCX) [file pmed.1003228.s006.docx]

**S4 Table. Treatment effect of bariatric surgery on body mass index (BMI)**

| **Bariatric operation** | **BMI after surgery (kg/m^2^)** | **Deterministic sensitivity analysis range** | **Probabilistic sensitivity analysis distribution** |
| --- | --- | --- | --- |
| Gastric bypass | 36.6 | +/-20% | Beta |
| Sleeve gastrectomy | 37.2 | +/-20% | Beta |
