# Supplementary material for: Bariatric surgery for patients with type 2 diabetes mellitus requiring insulin: Clinical outcome and cost-effectiveness analyses
Source: PLoS Med. 2020 Dec 7;17(12):e1003228. doi: 10.1371/journal.pmed.1003228 (PMC7721482; doi:10.1371/journal.pmed.1003228)
Supplement: S6 Table — (DOCX) [file pmed.1003228.s008.docx]

**S6 Table. Treatment effect of BMT on HbA1c**

| **Years from baseline** | **HbA1c (%)** | **Deterministic sensitivity analysis range** | **Probabilistic sensitivity analysis distribution** |
| --- | --- | --- | --- |
| Year 1 | 8.07 | +/-20% | Normal invariant on HbA1c changes from baseline |
| Year 2 | 8.15 |  |  |
| Year 3 | 8.20 |  |  |
| Year 4 | 8.41 |  |  |
| Year 5 | 8.36 |  |  |
