# Supplementary material for: Bariatric surgery for patients with type 2 diabetes mellitus requiring insulin: Clinical outcome and cost-effectiveness analyses
Source: PLoS Med. 2020 Dec 7;17(12):e1003228. doi: 10.1371/journal.pmed.1003228 (PMC7721482; doi:10.1371/journal.pmed.1003228)
Supplement: S7 Table — (DOCX) [file pmed.1003228.s009.docx]

**S7 Table. Mid-term bariatric surgery complications**

| **Mid-term surgery complications** | **Incidence 1^st^ year** | **Incidence 2^nd^ year** | **Deterministic sensitivity analysis range** | **Probabilistic sensitivity analysis distribution** |
| --- | --- | --- | --- | --- |
| Biliary disease requiring cholecystectomy | 2.88% | 1.19% | +/-20% | Beta |
| Abdominal wall herniae requiring surgery | 0.61% | 1.39% |  |  |
| Leakage and abscess | 0.22% | 0.15% |  |  |
| Stricture | 0.24% | 0.15% |  |  |
| Gastric ulcer | 1.12% | 1.07% |  |  |
| Obstruction | 1.73% | 3.19% |  |  |
