# Supplementary material for: Bariatric surgery for patients with type 2 diabetes mellitus requiring insulin: Clinical outcome and cost-effectiveness analyses
Source: PLoS Med. 2020 Dec 7;17(12):e1003228. doi: 10.1371/journal.pmed.1003228 (PMC7721482; doi:10.1371/journal.pmed.1003228)
Supplement: S8 Table — *% of patients with reduced drug dose and increase in HbA1c. TC:HDL (total cholesterol: high-density lipoproteins). (DOCX) [file pmed.1003228.s010.docx]

**S8 Table. Adverse drug events for surgical patients**

|  | **Cumulative adverse effects over 5 years** | **Deterministic sensitivity analysis range** | **Probabilistic sensitivity analysis distribution** |
| --- | --- | --- | --- |
| Oedema Risk (%) | 0 | Fixed | Fixed |
| Hypoglycemia Risk (%) | 3.94 | +/-20% | Beta |
| Hip Fracture Risk (%) | 0 | Fixed | Fixed |
| Non-Compliance Risk* | 0 | Fixed | Fixed |
| Weight gain (kg) | 0 | Fixed | Fixed |
| Deterioration in TC: HDL (%) | 0 | Fixed | Fixed |

* % of patients with reduced drug dose and increase in HbA1c. TC:HDL (total cholesterol: high-density lipoproteins)
