# Supplementary material for: Bariatric surgery for patients with type 2 diabetes mellitus requiring insulin: Clinical outcome and cost-effectiveness analyses
Source: PLoS Med. 2020 Dec 7;17(12):e1003228. doi: 10.1371/journal.pmed.1003228 (PMC7721482; doi:10.1371/journal.pmed.1003228)
Supplement: S10 Table — (DOCX) [file pmed.1003228.s012.docx]

**S10 Table. Treatment acquisition costs in bariatric surgery group**

| **Surgery** | **Cost (£)** | **Deterministic sensitivity analysis** | **Probabilistic sensitivity analysis distribution** |
| --- | --- | --- | --- |
| Gastric bypass procedure | 5853 | +/-20% | Gamma |
| Sleeve gastrectomy procedure | 5117 | +/-20% | Gamma |
| **Drugs post bariatric surgery** | **Cost (£)** | **Deterministic sensitivity analysis** | **Probabilistic sensitivity analysis distribution** |
| Metformin (70%) | 29.5 | +/-20% | Gamma |
| Insulin + Metformin (30%) | 439 | +/-20% | Gamma |
| Weighted average (100%) | 152 | +/-20% | Gamma |
