# Supplementary material for: Bariatric surgery for patients with type 2 diabetes mellitus requiring insulin: Clinical outcome and cost-effectiveness analyses
Source: PLoS Med. 2020 Dec 7;17(12):e1003228. doi: 10.1371/journal.pmed.1003228 (PMC7721482; doi:10.1371/journal.pmed.1003228)
Supplement: S11 Table — DPP4 = dipeptidyl peptidase 4; GLP-1 RA = glucagon like peptide-1 receptor agonist; SGLT2 = sodium glucose transport protein 2. (DOCX) [file pmed.1003228.s013.docx]

**S11 Table. Treatment acquisition costs in BMT group**

| **Years from baseline** | **Drug regimen** | **Cost (£)** | **Deterministic sensitivity analysis** | **Probabilistic sensitivity analysis distribution** |
| --- | --- | --- | --- | --- |
| Year 1 | Insulin + Metformin + DPP4 inhibitors | 1,525 | +/-20% | Gamma |
| Year 2 | Insulin + Metformin + GLP-1 RA | 2,283 | +/-20% | Gamma |
| Year 3 | Insulin + Metformin + SGLT2 inhibitor + GLP-1 RA | 2,759 | +/-20% | Gamma |
| Year 4 | Insulin + Metformin + SGLT2 inhibitor + GLP-1 RA | 2,759 | +/-20% | Gamma |
| Year 5 | Insulin + Metformin + SGLT2 inhibitor + GLP-1 RA | 2,759 | +/-20% | Gamma |

DPP4 = dipeptidyl peptidase 4; GLP-1 RA = glucagon like peptide-1 receptor agonist; SGLT2 = sodium glucose transport protein 2
