# Supplementary material for: Bariatric surgery for patients with type 2 diabetes mellitus requiring insulin: Clinical outcome and cost-effectiveness analyses
Source: PLoS Med. 2020 Dec 7;17(12):e1003228. doi: 10.1371/journal.pmed.1003228 (PMC7721482; doi:10.1371/journal.pmed.1003228)
Supplement: S12 Table — (DOCX) [file pmed.1003228.s014.docx]

**S12 Table.** **Cost of bariatric surgery complications**

| **Complication** | **Cost per event (£)** | **Deterministic sensitivity analysis** | **Probabilistic sensitivity analysis distribution** |
| --- | --- | --- | --- |
| Cholecystectomy, year 1 | 3722 | +/-20% | Gamma |
| Cholecystectomy, year 2 | 3722 | +/-20% | Gamma |
| Abdominal wall hernia operations, year 1 | 4353 | +/-20% | Gamma |
| Abdominal wall hernia operations, year 2 | 4353 | +/-20% | Gamma |
| Leakage and abscess, year 1 | 4456 | +/-20% | Gamma |
| Leakage and abscess, year 2 | 4456 | +/-20% | Gamma |
| Obstruction, year 1 | 2882 | +/-20% | Gamma |
| Obstruction, year 2 | 2882 | +/-20% | Gamma |
| Stricture, year 1 | 1760 | +/-20% | Gamma |
| Stricture, year 2 | 1760 | +/-20% | Gamma |
| Gastric ulcer, year 1 | 289 | +/-20% | Gamma |
| Gastric ulcer, year 2 | 289 | +/-20% | Gamma |
