# Supplementary material for: Bariatric surgery for patients with type 2 diabetes mellitus requiring insulin: Clinical outcome and cost-effectiveness analyses
Source: PLoS Med. 2020 Dec 7;17(12):e1003228. doi: 10.1371/journal.pmed.1003228 (PMC7721482; doi:10.1371/journal.pmed.1003228)
Supplement: S13 Table — *In-hospital costs of treating acute stroke. Weighted average of HRG AA35A, AA35B, AA35C, AA35D, AA35E, AA35F &Average of cost for years 2–5. ^Non-complication costs of treating T2DM. (DOCX) [file pmed.1003228.s015.docx]

**S13 Table.** **Costs of T2DM complications**

| **T2DM complication** | **Cost (£)** | **Deterministic sensitivity analysis** | **Probabilistic sensitivity analysis distribution** |
| --- | --- | --- | --- |
| Hypoglycaemia | 421 | +/-20% | Gamma |
| Ischemic Heart Disease at diagnosis year | 11203 | +/-20% | Gamma |
| Ischemic Heart Disease subsequent years | 1970 | +/-20% | Gamma |
| Acute myocardial infarction | 7737 | +/-20% | Gamma |
| Myocardial Infarction subsequent years | 1924 | +/-20% | Gamma |
| Congestive Heart Failure | 4394 | +/-20% | Gamma |
| Congestive Heart Failure subsequent years | 2577 | +/-20% | Gamma |
| Amputation | 12904 | +/-20% | Gamma |
| Amputation subsequent years | 3586 | +/-20% | Gamma |
| Blindness | 3314 | +/-20% | Gamma |
| Blindness subsequent years | 1255 | +/-20% | Gamma |
| Renal failure | 19242 | +/-20% | Gamma |
| Renal failure in subsequent years | 19242 | +/-20% | Gamma |
| Macrovascular Monitoring | 489 | +/-20% | Gamma |
| Macrovascular Monitoring in subsequent years | 401 | +/-20% | Gamma |
| Microvascular Monitoring | 424 | +/-20% | Gamma |
| Microvascular Monitoring in subsequent years | 317 | +/-20% | Gamma |
| Myocardial Infarction (fatal) | 1603 | +/-20% | Gamma |
| Stroke (fatal) | 4167 | +/-20% | Gamma |
| Acute stroke* | 5761 | +/-20% | Gamma |
| Stroke (non-fatal) 1st year | 9032 | +/-20% | Gamma |
| Stroke (non-fatal) subsequent years^&^ | 2316 | +/-20% | Gamma |
| Other^^^ | 3122 | +/-20% | Gamma |

*In-hospital costs of treating acute stroke. Weighted average of HRG AA35A, AA35B, AA35C, AA35D, AA35E, AA35F ^&^Average of cost for years 2-5. ^^^Non-complication costs of treating T2DM
