# Supplementary material for: Bariatric surgery for patients with type 2 diabetes mellitus requiring insulin: Clinical outcome and cost-effectiveness analyses
Source: PLoS Med. 2020 Dec 7;17(12):e1003228. doi: 10.1371/journal.pmed.1003228 (PMC7721482; doi:10.1371/journal.pmed.1003228)
Supplement: S14 Table — (DOCX) [file pmed.1003228.s016.docx]

**S14 Table. Utilities and decrements associated with individual complications of T2DM**

|  | **Decrement in utility value** | **Deterministic sensitivity analysis** | **Probabilistic sensitivity analysis distribution** |
| --- | --- | --- | --- |
| Ischaemic Heart Disease | -0.042 | -0.058 - 0.027 | Gamma |
| Myocardial Infarction (non-fatal) 1st year | -0.0626 | -0.07 - -0.05 | Gamma |
| Myocardial Infarction (non-fatal) subsequent years | -0.0368 | -0.04 - -0.03 | Gamma |
| Stroke (non-fatal) 1st year | -0.1171 | -0.14 - -0.03 | Gamma |
| Stroke (non-fatal) subsequent years | -0.0349 | -0.04 - -0.03 | Gamma |
| Congestive Heart Failure | -0.1167 | -0.14 - -0.09 | Gamma |
| Amputation | -0.095 | -0.173 - -0.018 | Gamma |
| Blindness | -0.045 | -0.062 - -0.027 | Gamma |
| Renal failure | -0.038 | -0.059 - -0.016 | Gamma |
| Oedema | -0.033 | -0.042 - -0.024 | Gamma |
| Fracture | -0.068 | -0.082 - -0.054 | Gamma |
| Hypoglycaemia | -0.047 | +/-20% | Gamma |
| Peripheral Vascular Disease | -0.045 | -0.071 - -0.019 | Gamma |
| Hypertension | -0.0375 | +/-20% | Gamma |
| Gastric bypass disutility | -0.21 | +/-20% | Gamma |
| Sleeve gastrectomy disutility | -0.21 | +/-20% | Gamma |
