# Supplementary material for: Bariatric surgery for patients with type 2 diabetes mellitus requiring insulin: Clinical outcome and cost-effectiveness analyses
Source: PLoS Med. 2020 Dec 7;17(12):e1003228. doi: 10.1371/journal.pmed.1003228 (PMC7721482; doi:10.1371/journal.pmed.1003228)
Supplement: S15 Table — (DOCX) [file pmed.1003228.s017.docx]

**S15 Table. Decrements in utility associated with bariatric surgery and body mass index (BMI) category**

| **Decrements in utility associated with therapy and BMI category** | **Value** | **Deterministic sensitivity analysis** | **Probabilistic sensitivity analysis distribution** |
| --- | --- | --- | --- |
| Gastric bypass disutility | -0.21 | +/-20% | Gamma |
| Sleeve gastrectomy disutility | -0.21 | +/-20% | Gamma |
| BMI category 30–34 kg/m^2^ | -0.085 | +/-20% | Gamma |
| BMI category 35–39 kg/m^2^ | -0.17 | +/-20% | Gamma |
| BMI category ≥40 kg/m^2^ | -0.255 | +/-20% | Gamma |
