# Supplementary material for: Bariatric surgery for patients with type 2 diabetes mellitus requiring insulin: Clinical outcome and cost-effectiveness analyses
Source: PLoS Med. 2020 Dec 7;17(12):e1003228. doi: 10.1371/journal.pmed.1003228 (PMC7721482; doi:10.1371/journal.pmed.1003228)
Supplement: S16 Table — *% of patients with event over 5 years. (DOCX) [file pmed.1003228.s018.docx]

**S16 Table. Additional model results**

| **Outcomes** | **Bariatric surgery (BS)** | **Best medical treatment (BMT)** | **Incremental difference**  **(BS-BMT)** |
| --- | --- | --- | --- |
| Adverse event (excluding death)* | 14 | 20 | -6 |
| Diabetes related deaths* | 4.97 | 6.71 | -1.74 |
| Other deaths* | 5.25 | 5.58 | -0.33 |
| Life Years gained | 4.47 | 4.43 | 0.04 |

*% of patients with event over 5 years
