# Supplementary material for: Bariatric surgery for patients with type 2 diabetes mellitus requiring insulin: Clinical outcome and cost-effectiveness analyses
Source: PLoS Med. 2020 Dec 7;17(12):e1003228. doi: 10.1371/journal.pmed.1003228 (PMC7721482; doi:10.1371/journal.pmed.1003228)
Supplement: S17 Table — (DOCX) [file pmed.1003228.s019.docx]

**S17 Table. Probabilistic sensitivity analysis results (1,000 iterations)**

| **Outcomes** | **Bariatric surgery (BS)** | | **Best medical treatment (BMT)** | | **Incremental difference**  **(BS-BMT)** |
| --- | --- | --- | --- | --- | --- |
|  | **Mean** | **95% CI** | **Mean** | **95% CI** |  |
| Total expected costs (£) | 22,291 | 21993.4 - 22619.6 | 26,668 | 26367.1 - 27031.85 | - 4,377 |
| Total expected QALYs | 3.485 | 3.46 - 3.52 | 3.469 | 3.43 - 3.5 | 0.0155 |
