# Supplementary material for: Bariatric surgery for patients with type 2 diabetes mellitus requiring insulin: Clinical outcome and cost-effectiveness analyses
Source: PLoS Med. 2020 Dec 7;17(12):e1003228. doi: 10.1371/journal.pmed.1003228 (PMC7721482; doi:10.1371/journal.pmed.1003228)
Supplement: S18 Table — *% of patients with event over 5 years. (DOCX) [file pmed.1003228.s020.docx]

**S18 Table. Cost-effectiveness results for Afro-Caribbean population**

| **Outcomes/Comparator** | **Bariatric surgery** | **Best medical treatment** |
| --- | --- | --- |
| Adverse health event (excluding death)* | 15% | 18% |
| Diabetes-related deaths* | 1.68% | 1.94% |
| Other deaths* | 5.64% | 5.80% |
| Average Total costs (£) | 22,381 | 26,853 |
| Drug costs (£) | 6,551 | 10,810 |
| Cost of complications (£) | 15,019 | 15,069 |
| Adverse Event Costs (£) | 811 | 974 |
| Average QALYs | 3.52 | 3.51 |
| Life Years | 4.53 | 4.52 |
| Incremental Cost per QALY | Dominated by bariatric surgery | |
| Incremental cost per life-year gained | Dominated by bariatric surgery | |

*% of patients with event over 5 years
