# Supplementary material for: Bariatric surgery for patients with type 2 diabetes mellitus requiring insulin: Clinical outcome and cost-effectiveness analyses
Source: PLoS Med. 2020 Dec 7;17(12):e1003228. doi: 10.1371/journal.pmed.1003228 (PMC7721482; doi:10.1371/journal.pmed.1003228)
Supplement: S19 Table — *% of patients with event over 5 years. (DOCX) [file pmed.1003228.s021.docx]

**S19 Table. Cost-effectiveness results for** **Indian-Asian population**

| **Outcomes/Comparator** | **Bariatric surgery** | **Best medical treatment** |
| --- | --- | --- |
| Adverse event (excluding death)* | 19% | 22% |
| Diabetes related deaths | 3.48% | 4.37% |
| Other deaths | 5.27% | 5.07% |
| Average Total costs (£) | 22,263 | 26,735 |
| Drug costs (£) | 6,522 | 10,689 |
| Cost of complications (£) | 14,938 | 15,083 |
| Adverse Event Costs (£) | 803 | 964 |
| Average QALYs | 3.49 | 3.47 |
| Life Years | 4.49 | 4.48 |
| Incremental Cost per QALY | Dominated by bariatric surgery | |
| Incremental cost per life year gained | Dominated by bariatric surgery | |

*% of patients with event over 5 years
