# Supplementary material for: Bariatric surgery for patients with type 2 diabetes mellitus requiring insulin: Clinical outcome and cost-effectiveness analyses
Source: PLoS Med. 2020 Dec 7;17(12):e1003228. doi: 10.1371/journal.pmed.1003228 (PMC7721482; doi:10.1371/journal.pmed.1003228)
Supplement: S20 Table — (DOCX) [file pmed.1003228.s022.docx]

**S20 Table. Cost-effectiveness results when estimated annual rate of hypoglycaemia in BMT group is constant at 2.43% across 5 years**

|  | **Bariatric surgery (BS)** | **Best medical treatment (BMT)** |  |
| --- | --- | --- | --- |
| Hypoglycaemia cost (£/year) | 57 | 44 |  |
| Monitoring costs (£/year) | 744 | 949 |  |
|  | **Bariatric surgery (BS)** | **Best medical treatment (BMT)** | **Incremental difference (BS-BMT)** |
| Average total costs (£) | 22,019 | 26,471 | - 4,453 |
| Treatment costs (£) | 6,172 | 10,629 | - 4,457 |
| Cost of complications (£) | 14,697 | 14,850 | - 153 |
| Adverse Event Costs (£) | 1,150 | 993 | 157 |
| Average QALYs | 3.18 | 3.15 | 0.03 |
| Life Years | 4.47 | 4.43 | 0.04 |
