# Supplementary figures and images for: Bariatric surgery for patients with type 2 diabetes mellitus requiring insulin: Clinical outcome and cost-effectiveness analyses
Source: PLoS Med. 2020 Dec 7;17(12):e1003228. doi: 10.1371/journal.pmed.1003228 (PMC7721482; doi:10.1371/journal.pmed.1003228)

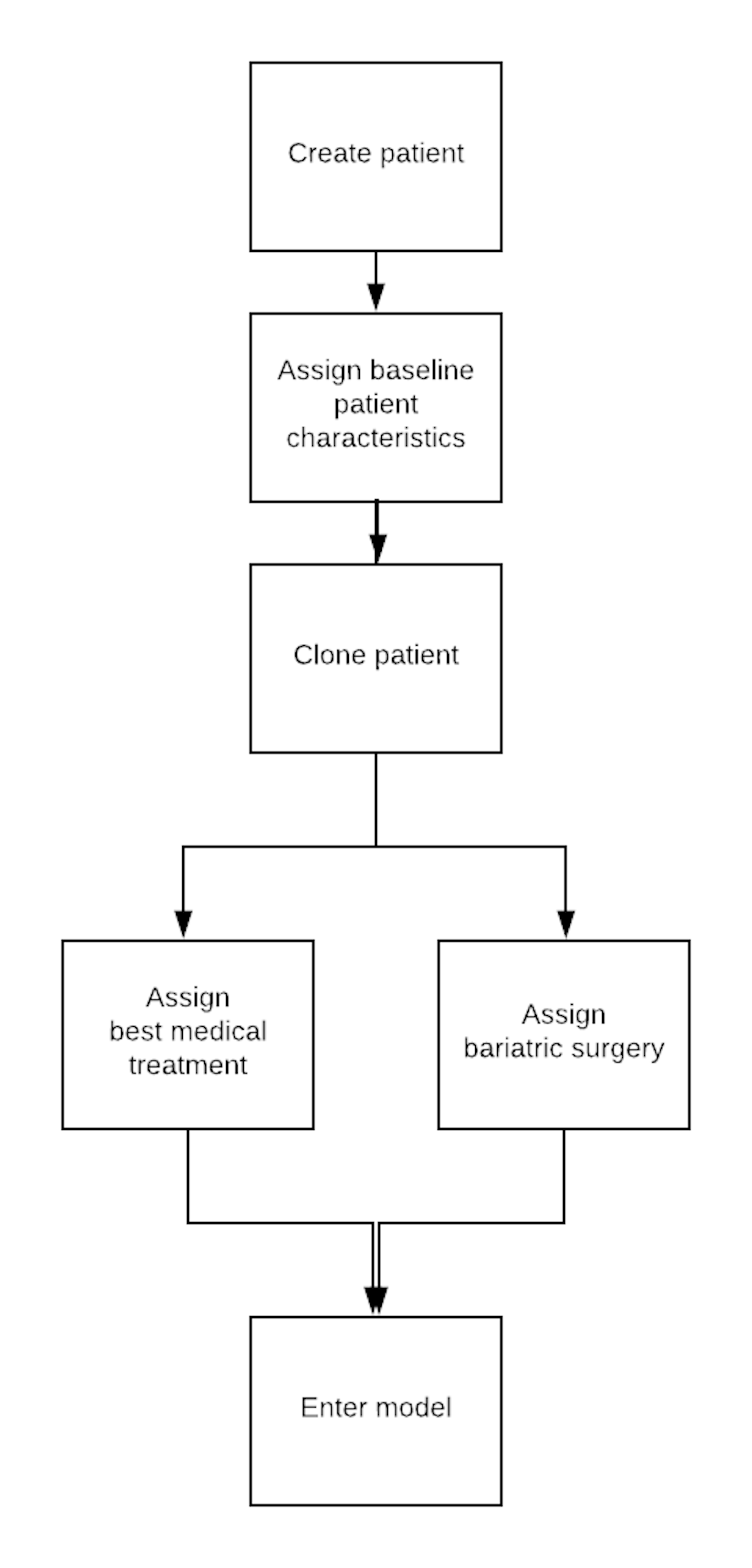

Supplement: S1 Fig — (TIF) [file pmed.1003228.s023.tif]

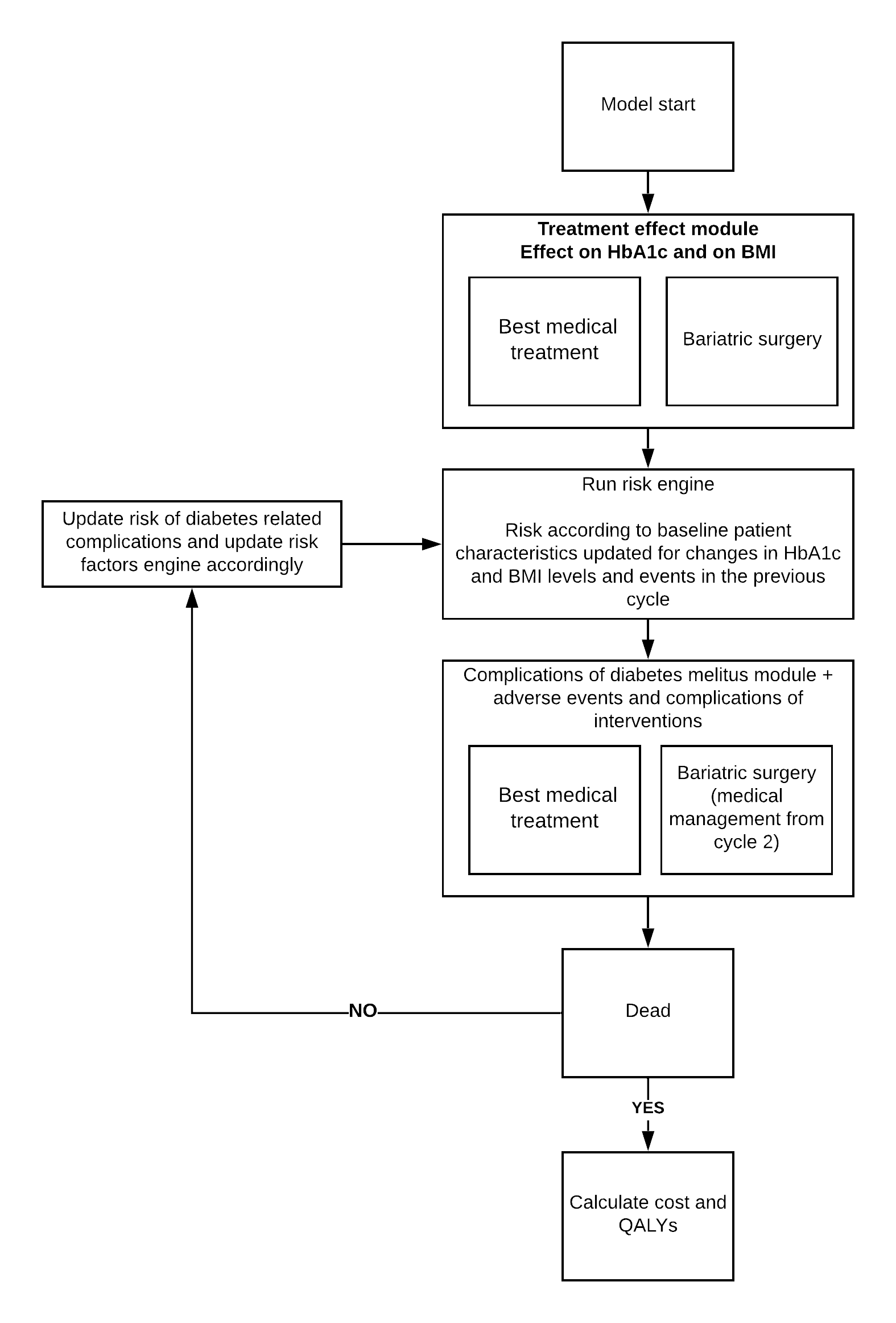

Supplement: S2 Fig — (TIF) [file pmed.1003228.s024.tif]

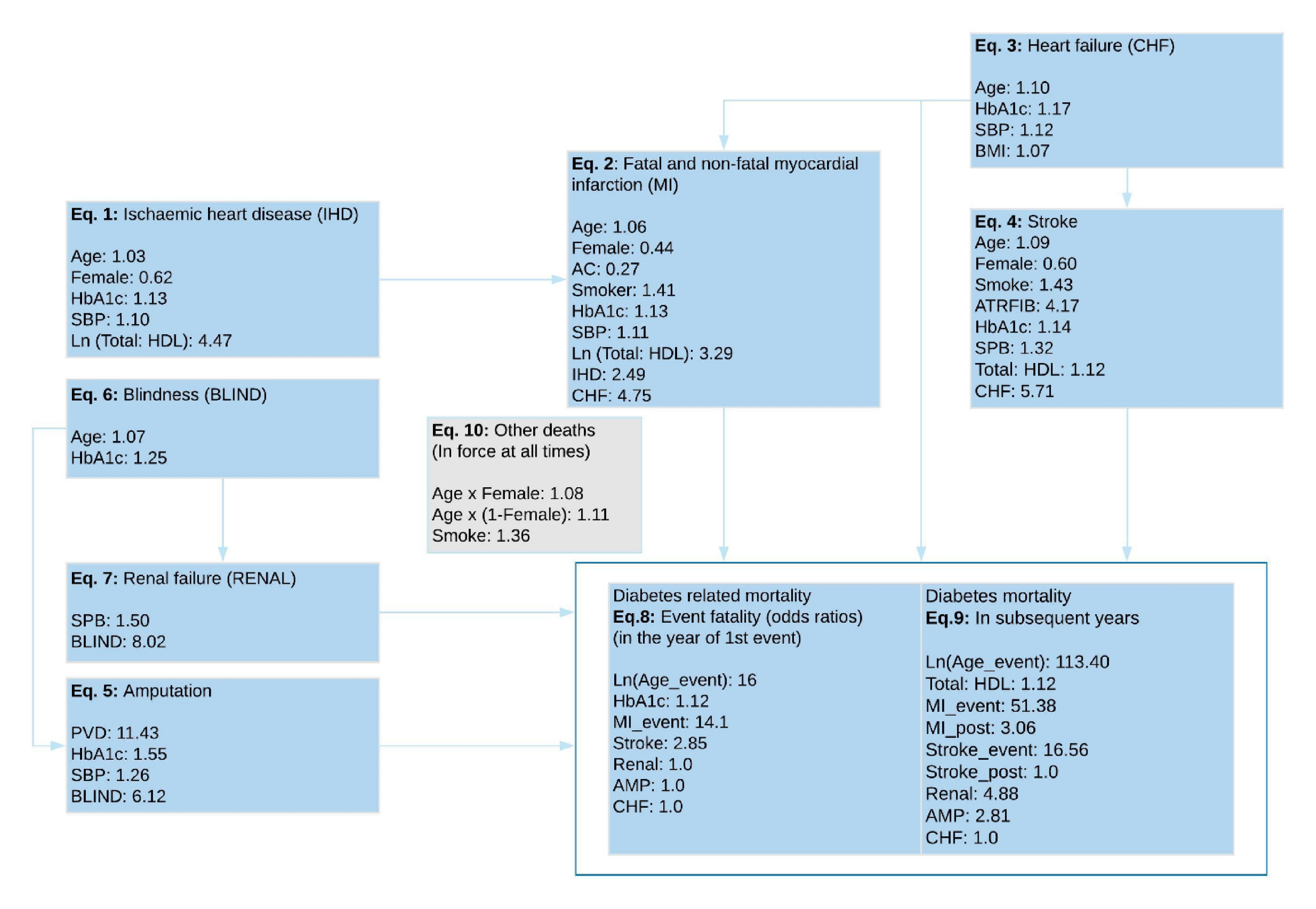

Supplement: S3 Fig — (TIF) [file pmed.1003228.s025.tif]

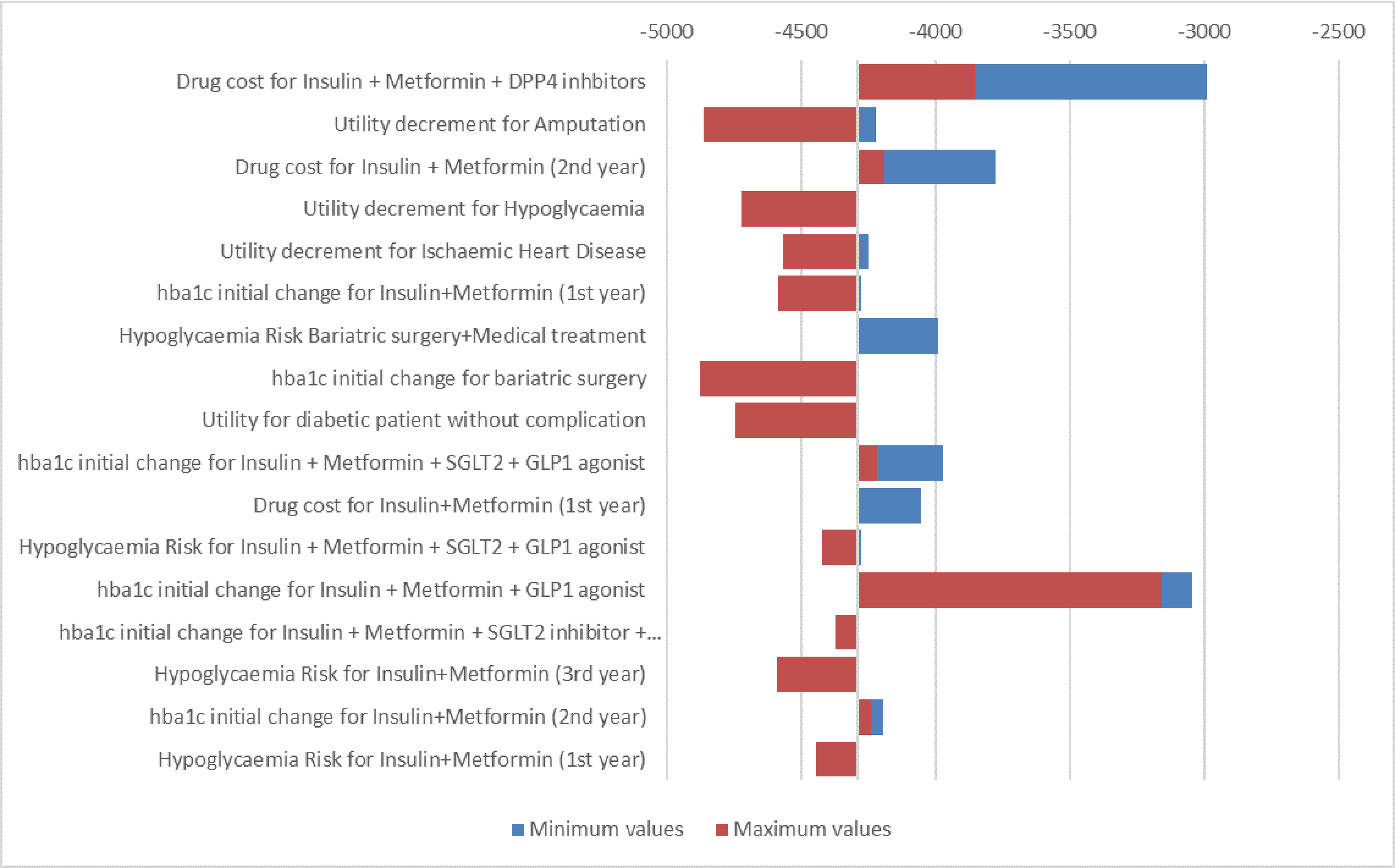

Supplement: S4 Fig — (TIF) [file pmed.1003228.s026.tif]

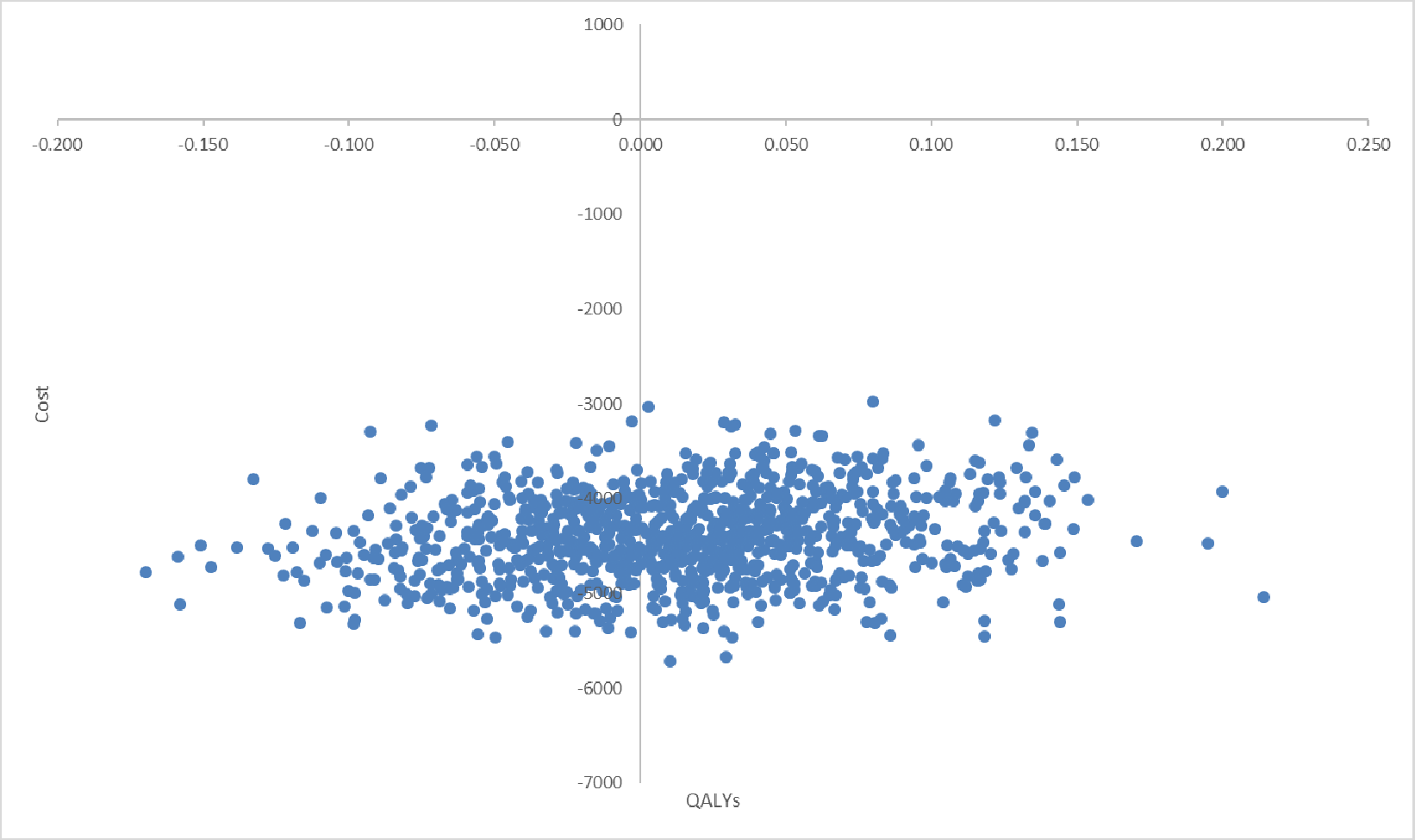

Supplement: S5 Fig — (TIF) [file pmed.1003228.s027.tif]
